# Base Editors-Mediated Gene Therapy in Hematopoietic Stem Cells for Hematologic Diseases

**DOI:** 10.1007/s12015-024-10715-5

**Published:** 2024-04-22

**Authors:** Chengpeng Zhang, Jinchao Xu, Yikang Wu, Can Xu, Peng Xu

**Affiliations:** Cyrus Tang Medical Institute, National Clinical Research Center for Hematologic Diseases, State Key Laboratory of Radiation Medicine and Protection, Collaborative Innovation Center of Hematology, Soochow Medical College, Soochow University, Suzhou, 215123 Jiangsu Province China

**Keywords:** Base editors, CRISPR/Cas system, Hematopoietic stem cells, Gene therapy

## Abstract

Base editors, developed from the CRISPR/Cas system, consist of components such as deaminase and Cas variants. Since their emergence in 2016, the precision, efficiency, and safety of base editors have been gradually optimized. The feasibility of using base editors in gene therapy has been demonstrated in several disease models. Compared with the CRISPR/Cas system, base editors have shown great potential in hematopoietic stem cells (HSCs) and HSC-based gene therapy, because they do not generate double-stranded breaks (DSBs) while achieving the precise realization of single-base substitutions. This precise editing mechanism allows for the permanent correction of genetic defects directly at their source within HSCs, thus promising a lasting therapeutic effect. Recent advances in base editors are expected to significantly increase the number of clinical trials for HSC-based gene therapies. In this review, we summarize the development and recent progress of DNA base editors, discuss their applications in HSC gene therapy, and highlight the prospects and challenges of future clinical stem cell therapies.

**Graphical Abstract:**

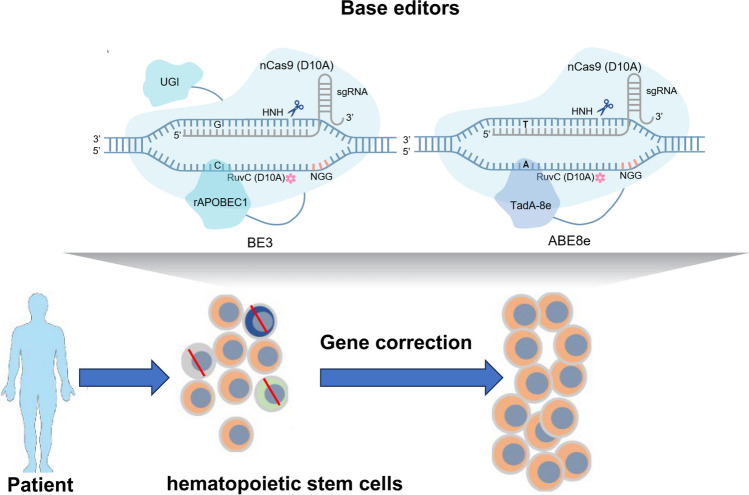

## Introduction

Hematopoietic stem cells (HSCs) proliferate over time and can potentially differentiate into various types of mature blood cells [1]. HSC transplantation (HSCT) replaces diseased HSCs with normal HSCs and is widely used to treat a variety of blood disorders, including leukemia, lymphoma, sickle cell disease (SCD), and thalassemia [2]. HSCT can be divided into allogeneic HSCT and autologous HSCT. Allogeneic HSCT has a certain probability of inducing graft-versus-host disease (GVHD) through immune rejection, which can negatively affect the health of patients [3]. In contrast, autologous HSCT, which is achieved through gene editing, is considered a safer strategy. Numerous clinical studies are currently in progress, and the effectiveness of HSC-based gene therapy has been effectively verified in several animal models through the development of gene editing technologies such as the CRISPR/Cas system and base editors. Significantly, the first CRISPR/Cas9-mediated HSC gene editing drug was approved by the FDA for SCD and β-thalassemia treatment [4].

**Fig. 1:**
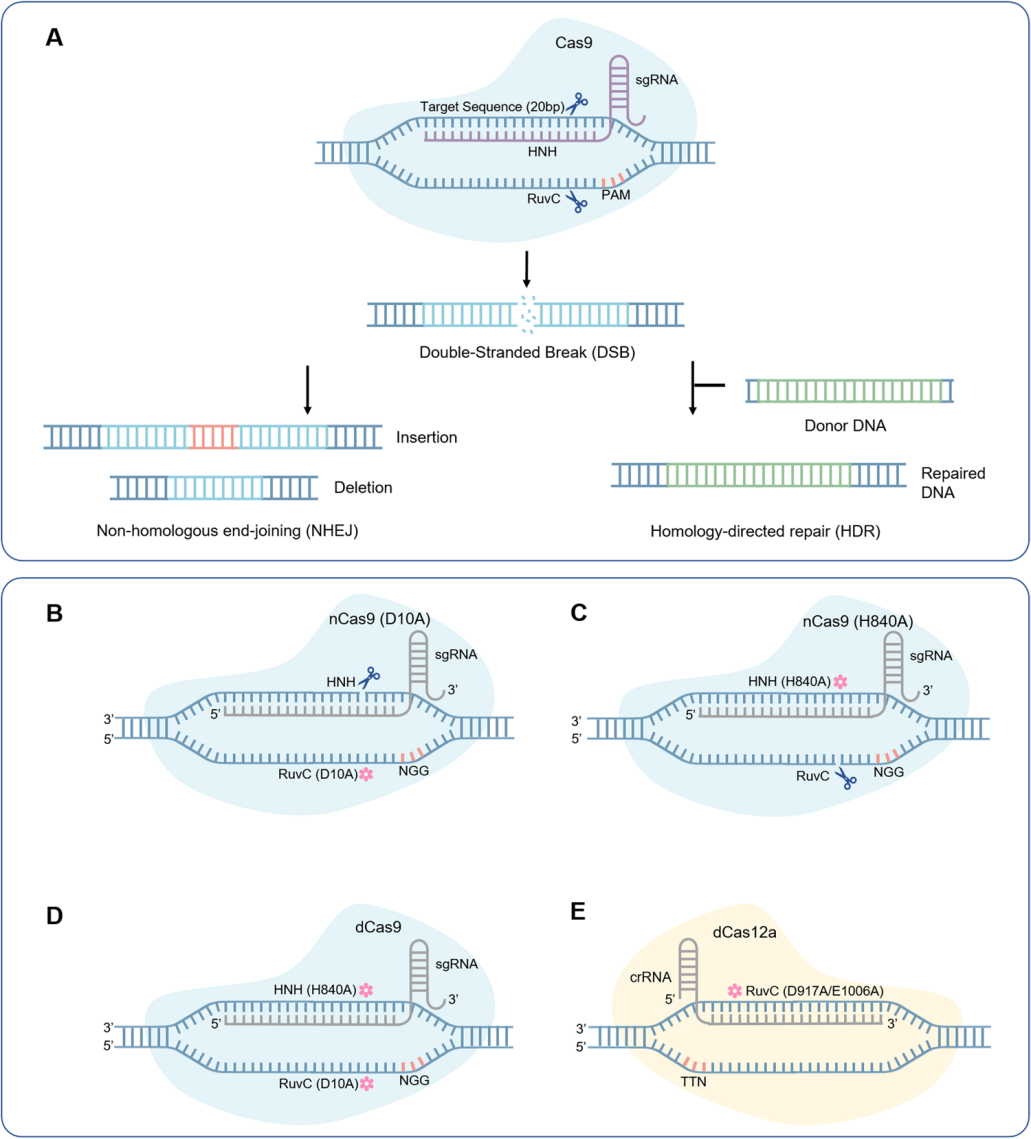
CRISPR/Cas system-mediated gene editing with four Cas variants. **a** CRISPR/Cas9 system-mediated gene editing for gene manipulation via different DNA repair pathways. **b** Introduction of a D10A single mutation in the RuvC structural domain of Cas9 to obtain Cas9 nickase (nCas9 (D10A)). **c** Introduction of an HB40A single mutation in the HNH structural domain of Cas9 to obtain nCas9 (H840A). **d** Introduction of D10A and HB40A double mutations in the RuvC and HNH structural domains of Cas9, respectively, to obtain catalytically dead Cas9 (dCas9). **e** Introduction of D917A or E1006A single mutations in the RuvC structural domain of Cas12a to obtain dCas12a

The CRISPR/Cas system consists of the clustered regularly interspaced short palindromic repeat (CRISPR) and its associated protein (Cas). This system is the third generation of gene-editing systems, after zinc finger nuclease (ZFN) and transcription activator-like effector nuclease (TALEN) [5]. CRISPR/Cas9 is currently the most widely used of the CRISPR/Cas systems. The basic principle of CRISPR/Cas9 is the construction of a ribonucleoprotein complex with two key components, single guide RNA (sgRNA) and the Cas9 nuclease, to cause a double-stranded break (DSB) at a specific DNA location [6]. There are two ways to repair genomic DNA after DSBs: homology-directed repair (HDR) and nonhomologous end-joining (NHEJ) (Fig. 1a) [7]. Despite the enormous potential of the CRISPR/Cas9 system, it still has certain limitations and drawbacks, including the dependence of HDR on dividing cells, the poor precision of NHEJ, and the generation of random DNA insertion or deletion mutations (indels) [8, 9]. Therefore, new generations of precise gene editing technology beyond CRISPR/Cas9 are urgently needed.

**Fig. 2:**
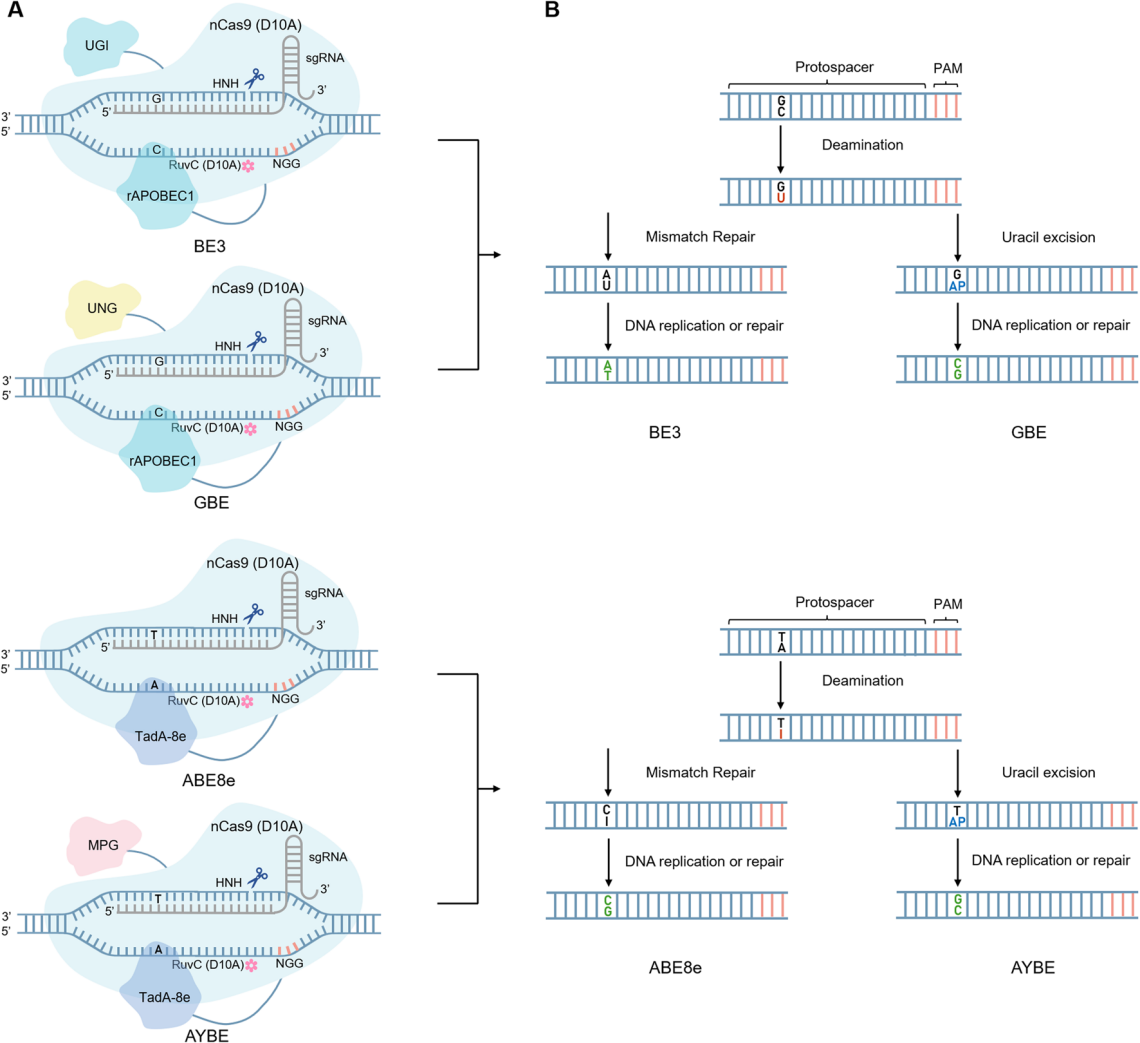
Overview of different base editors. **a** BE3 is capable of the C-G to T-A transition. GBE is capable of C-G to G-C transversion. ABE8e is capable of the A-T to G-C transition. AYBE is capable of A-T to C-G transversion. **b** Mechanisms of action of BE3, GBE, ABE8e and AYBE. For both BE3 and GBE, the cytidine deaminase rAPOBEC1 enables the deamination of G-C to G-U. Then, the DNA undergoes G-U to A-U mismatch repair since UGI of BE3 inhibits UNG activity. Finally, in DNA repair or replication, T replaces U to realize the desired C-G to T-A transition. In contrast, after deamination, the UNG contained in GBE can excise U to form AP sites, which can lead to a variety of base pairing events, resulting in the desired C-G to G-C transversion in subsequent DNA repair or replication. For ABE8e and AYBE, the adenine deaminase TadA-8e enables the deamination of T-A to T-I. Then, the DNA undergoes T-I to C-I mismatch repair. Finally, in DNA repair or replication, G replaces I to realize the desired A-T to G-C transition. Above is the mechanism of ABE8e. AYBE, on the other hand, contains an MPG that can excise I to form AP sites after deamination, which can lead to a variety of base pairing events, resulting in the desired A-T to C-G and A-T to T-A transversions in subsequent DNA repair or replication

Base editors are more precise gene editing tools that were developed based on the CRISPR/Cas9 system. By combining the catalytic activity of base deaminase with the targeting specificity of the CRISPR/Cas system to substitute specific bases in the target gene, base editors can achieve single-base editing with a high degree of precision without causing DSBs [10]. Recent studies have developed a variety of base editors, including cytosine base editors (CBEs), capable of C-G to T-A transitions; adenosine base editors (ABEs), capable of A-T to G-C transitions; glycosylase base editors (GBEs), capable of C-G to G-C transversions; and adenine transversion editors, capable of A-T to C-G transversions (Fig. 2a) [10–13]. Compared with the CRISPR/Cas system, base editors have greater editing efficiency and lower off-target activity and thus have great potential in gene therapy and disease research. In addition to base editors, prime editors (PEs) are another type of gene editing tool. Compared to base editors, PEs can mediate targeted insertions, deletions, and all 12 possible base-to-base transitions in human cells [14]. Although PEs have caused substantial progress in the study of disease models, the editing efficiency, complexity, and payload size of PEs are still major challenges in gene therapy [15].

In this review, we focused on the advances in base editing technology-mediated HSC gene therapy. Given the encouraging progress of CRISPR/Cas9-mediated gene therapy for SCD and β-thalassemia, we subsequently discussed the potential applications of base editing technology-mediated HSC gene therapy in different types of inherited hematologic diseases. Finally, long-term safety is a major concern for all gene editing methods used as therapeutic strategies. Therefore, we briefly discussed the potential off-target editing events of base editing technology.

## Overview of Base Editors

### From the CRISPR/Cas System to Base Editors

The CRISPR/Cas system, a widely used gene editing tool, often generates indel byproducts during genome editing because the system relies on the DNA repair pathway to achieve the integration, knockout, and replacement of genes. To solve this problem, the base editor, a gene editing tool that does not induce DSBs, has been developed. The main CRISPR/Cas systems currently used for base editors are the Type II CRISPR/Cas9 system and the Type V CRISPR/Cas12a system.

The CRISPR/Cas9 system consists of the Cas9 nuclease, CRISPR RNA (crRNA), and transactivating crRNA (tracrRNA) [16, 17]. The Cas9 nuclease recognizes the protospacer adjacent motif (PAM), which is located downstream of the 3' end of the protospacer on the nontarget DNA strand [18]. Subsequently, the crRNA and tracrRNA combine to form a sgRNA, which guides the Cas9 nuclease to achieve DNA cleavage through complementary base pairing. The CRISPR/Cas12a system consists of the Cas12a nuclease and crRNA. The Cas12a nuclease recognizes the PAM upstream of the 5' end of the nontarget DNA strand and is guided to cleave double-stranded DNA by mature crRNA [19, 20].

To reduce the production of indels and decrease cytotoxicity, different point mutations were created on Cas9 and Cas12a. Single point mutations (D10A or H840A) and double point mutations (D10A and H840A) were introduced into the two active nuclease structural domains (RuvC and HNH) of Cas9 to obtain Cas9 nickase (nCas9) (Fig. 1b and c) and catalytically dead Cas9 (dCas9) (Fig. 1d), respectively [6, 21]. nCas9 cleaves single-stranded DNA (ssDNA) to form incisions, while the nuclease activity of dCas9 disappears; however, both proteins still utilize sgRNA to direct their binding to DNA. Similarly, introducing a point mutation (D917A or E1006A) into the RuvC active nuclease structural domain of wild-type Cas12a results in dCas12a with an inactivated DNA cutting function (Fig. 1e) [22, 23]. To achieve more precise gene editing, Komor et al. [10] developed the first generation of cytosine base editor (BE1) by expressing dCas9 fused with the rat cytosine deaminase rAPOBEC1. Since this development, base editors have entered the stage of gene editing, and various versions of base editors have provided new strategies for gene therapy. In this section, we provide a brief overview of the mechanisms of different kinds of base editors and their advancement (Fig. 2b).

### Cytosine Base Editors

The first-generation cytosine base editor (BE1) consists of the rat cytosine deaminase rAPOBEC1 and dCas9 joined by the flexible linker protein XTEN to form the rAPOBEC1-XTEN-dCas9 fusion protein. Through the dual action of complementary pairing of sgRNA and protospacer sequences and recognition of PAM sequences, dCas9 denatures the DNA, resulting in the formation of the dCas9-sgRNA-DNA R-loop complex, which enables the action of ssDNA cytosine deaminase to catalyze the deamination of cytosine (C) on the nontarget DNA strand to uracil (U). Uracil is analogous to thymine (T) during the DNA replication or repair process, serving as a complement to adenine (A) and ultimately facilitating the C-G to T-A transition. However, U-G to C-G repair by intracellular uracil N-glycosylase (UNG) resulted in inefficient editing of BE1 in HEK293T cells, ranging from 0.8% to 7.7%. Therefore, uracil DNA glycosylase inhibitor (UGI) from the *B. subtilis* bacteriophage PBS1 was introduced in BE2 to inhibit intracellular UNG activity, and the average base editing efficiency of BE2 in HEK293T cells was three times greater than that of BE1. BE3 is built on BE2 by replacing dCas9 with nCas9 (D10A), which cleaves the strand at a guanine (G) residue to form an incision, inducing a mismatch repair (MMR) mechanism to achieve U-G to U-A repair. The average base editing efficiency of BE3 is two to six times greater than that of BE2 [10].

The C-G to T-A transition achieved by CBEs can be accompanied by a C-G to non-T-A transition and the production of a small number of detectable indels, reducing the purity of the edited product. To improve purity, Komor et al. [24] constructed a fourth-generation cytosine base editor, BE4, by fusing a second UGI to BE3 and adjusting the length of the linker protein; BE4 better inhibited the activity of intracellular UNG and improved the efficiency of base editing and the purity of the product. Compared to that with BE3, the production of non-T bases with BE4 was reduced by an average of 2.3 ± 0.3-fold, and the frequency of indel production was reduced by an average of 2.3 ± 1.1-fold.

An activity window is the specific region of DNA where a base editor performs base editing, usually defined as a few nucleotides (nt) away from the PAM sequence [25]. For example, BE3 has an activity window of approximately 5 nt, and if the PAM sequence is numbered 21 to 23, the activity window is numbered 4 to 8 [10]. Editing bases outside the activity window is less efficient. Bystander editing likely occurs when a nontarget base is within the active window [26]. To reduce bystander editing, researchers have developed a variety of base editors with narrower windows of activity. Chen et al. [27] constructed Td-CBEs by introducing the N46L mutation to the adenine deaminase TadA-8e so that only cytosine was used as a substrate for deamination. Of these Td-CBEs, eTd-CBEm edits only cytosines at position 5, with the activity window narrowing to 1 nt, greatly preventing bystander editing. Similarly, Neugebauer et al. [28] used phage-assisted continuous evolution (PACE) to evolve TadA-8e to obtain TadA-CDs, which also only use cytosine as a substrate, and constructed a series of small and highly efficient TadCBEs that retain the characteristics of ABEs.

Notably, a variety of methods, including direct evolution, PACE, and artificial intelligence (AI) tools, were employed in the generation and optimization of the base editors, which may provide further insight into the future development of base editing [28–36]. Therefore, they are specifically summarized (Table 1).

**Table 1:** Methods and resources involved in the development of base editors

Methods	Features	Target components	Modification examples	Aim	References
Biochemical screen for Cas9 orthologues	Construction of plasmid DNA libraries allows rapid determination of the PAM sequence of each Cas9 orthologue	Cas nucleases	SaCas9、Nme2Cas9、LbdCas12a	Expanding PAM Compatibility	[30, 31]
Direct evolution	Re-engineering the substrate specificity of proteins from a diverse range of families	Cas nucleases	Sp nCas9 (D10A/D1135V/R1335Q/T1337R); Sp nCas9 (D10A/D1332K/R1333G)	Expanding PAM Compatibility	[35, 36]
PACE	Enabling the rapid continuous evolution of biomolecules through many generations of mutation, selection, and replication	Cas nucleases	dxCas9 (3.7) (D10A/A262T/R324L/S409I/E480K/E543D/M694I/H840A/E1219V)	Expanding PAM Compatibility	[34]
		Deaminases	TadA-8e、TadA-CDs	Improving deamination activity	[28, 32]
PANCE	Sharing the same selection principles as PACE but is performed through serial dilution instead of under continuous flow	Deaminases	TadA-8e、TadA-CDs	Improving deamination activity	[28, 32]
Site-directed mutagenesis	Enhancing target protein traits rapidly and effectively	Deaminases	YE1 APOBEC1 (W90Y, R126E) YE2 APOBEC1 (W90Y, R132E)	Minimizing bystander editing	[33]
AI-guided structure-based protein clustering	Utilizing AlphaFold2 to predict and subsequently cluster an entire protein family based on predicted structure similarities	Deaminases	Sdd-CBEs	Identifying new properties of deaminases	[29]

### Adenine Base Editors

Approximately 14% of all human pathogenic single-nucleotide variations (SNVs) can be corrected through a C-G to T-A transition, which can be achieved by CBEs. However, approximately 48% of SNVs require the A-T to G-C transition for correction [37]. To address this problem, Gaudelli et al. [11] developed ABEs that enable the A-T to G-C transition. Adenosine can be deaminated to inosine, which is analogous to guanosine and paired with cytosine during repair or replication. On this basis, researchers have tried to test the editing effect of natural adenosine deaminase by fusing it with nCas9. However, since none of the natural adenosine deaminases act on ssDNA, the A-T to G-C transition was not achieved. Therefore, to obtain an adenosine deaminase that acts on ssDNA, the team evolved a transfer RNA (tRNA) adenosine deaminase enzyme (TadA) from *E. coli*. After seven evolutionary cycles, ABE7.10 was obtained; this protein consists of the heterodimer wtTadA-TadA* (wtTadA is the wild-type TadA, and TadA* contains 14 amino acid mutant sites), which is fused to nCas9, and it exhibited an average editing efficiency of 53 ± 3.7% across 17 tested gene loci in HEK293T cells [11]. Koblan et al. [38] constructed ABEmax by fusing the bpNLS at both ends of ABE7.10 in place of the SV40 NLS; ABEmax exhibited even greater editing efficiency.

To further improve adenosine deaminase activity, Richter et al. [32] used phage-assisted noncontinuous evolution (PANCE) and PACE techniques to evolve TadA-7.10, the deaminase component of ABE7.10, to obtain TadA-8e. Compared to TadA-7.10, TadA-8e contains eight additional mutations, which increase its activity by 590-fold. Researchers constructed four variants of ABE8e (SpABE8e, SaABE8e, LbABE8e, and enAsABE8e) based on four different Cas proteins (SpCas9, SaCas9, dLbCas12a, and enAsCas12a). Among them, SpABE8e exhibited the most obvious increase in base editing efficiency compared to ABE7.10, while the remaining three variants extended the editing scope and led to a substantial increase in editing efficiency [32].

### Glycosylase Base Editors

To develop base editors capable of C-G to G-C transversion, Kurt et al. [39] removed the UGI in BE4max, resulting in the construction of BE4max∆UGI, which allows for a slight increase in C-G to G-C transversion. Although UNG can excise uracil to form apurinic/apyrimidinic (AP) sites, subsequent DNA repair or replication can lead to a variety of editing results. Accordingly, the researchers hypothesized that fusion of human UNG enzymes could improve editing efficiency. However, the BE4max∆UGI-hUNG fusion product reduced the C-G to G-C editing activity. In response, researchers introduced the R33A mutation into rAPOBEC1 and constructed BE4max(R33A)∆UGI-hUNG, which improved editing efficiency and decreased indel production. The researchers further replaced the human UNG enzyme with the UNG enzyme from *E. coli* and fused it to the amino terminus to construct eUNG-BE4max(R33A)∆UGI and CGBE1. Of the 18 gene loci in human HEK293T cells, four showed efficient C-G to G-C editing activity, with an average editing frequency of 41.7–71.5%. Moreover, the researchers removed the eUNG structural domain in CGBE1 and constructed miniCGBE1, which had a smaller size. miniCGBE1 significantly reduced the indel generation rate while slightly decreasing the editing efficiency compared with CGBE1 [39].

In the same year, Zhao et al. [12] similarly developed GBEs capable of C-G to G-C transversion and C-G to A-T transversion. Among them, researchers have utilized AID in *E. coli* to construct AID-nCas9-UNG, which achieved an average editing efficiency of 87.2% ± 6.9% for C-G to A-T transversion. In mammalian cells, researchers constructed the rAPOBEC1-nCas9-UNG complex, which has a high editing efficiency at position 6 of the protospacer, ranging from 5.3% to 53.0% at 30 sites [12].

### Adenine Transversion Editors

Tong et al. [13] hypothesized that the base excision repair (BER) pathway could be induced in mammalian cells to achieve A-T to C-G and A-T to T-A transversion editing. The wild-type human N-methylpurine DNA glycosylase protein (MPG) can excise the hypoxanthine base (Hx) in deoxyinosine (I) formed by the deamination of adenine to form AP sites; this step is followed by DNA repair or replication, which can also lead to multiple editing events. Experimenters developed adenine transversion editors (AYBE, Y = C/T) by fusing ABE8e with MPG. First, the researchers fused MPG to the carbonyl terminus of ABE8e to construct AYBEv0.1, followed by the introduction of the N169S mutation to MPG to construct AYBEv0.2. Subsequently, the researchers performed two rounds of mutation screening in AYBEv0.2 for the presence of AYBEv1 with MPG-F8V1 as well as AYBEv1 with MPG-G163R and N169S of AYBEv2, both of which have high editing activity. Finally, to explore the synergistic effect of the AYBEv1 and AYBEv2 mutations, the researchers combined the mutations in AYBEv3. Compared with AYBEv0.1, the transversion editing activity of AYBEv3 was synergistically enhanced by 4.78-fold, with predominantly A-T to C-G transversion editing, and all transversion editing efficiencies reached up to 72%.

### Mitochondrial Base Editors

Mitochondria are critical for the differentiation and commitment of HSCs [40]. Increased mitochondrial DNA (mtDNA) mutations in HSCs may lead to delayed transferrin receptor (TfR) clearance, which results in increased free iron, erythrocyte cell membrane modification, and subsequently a decrease in erythrocyte lifespan, leading to mitochondrial anemia [41]. It has been shown that point mutations in the cytochrome coxidase subunit I (*MT-CO1*) gene can lead to the development of acquired idiopathic sideroblastic anemia [42]. Thus, base editing of mtDNA in HSCs is expected to be another target for the treatment of blood disorders. RNA-free DddA-derived cytosine base editors (DdCBEs) are the first mitochondrial base editors. Researchers have described an interbacterial toxin called DddA that mediates the deamination of dsDNA. DdCBEs consist of two nontoxic split-DddA halves, transcription activator-like effector (TALE) array proteins and a UGI, catalyzing the C-G to T-A transition in mtDNA [43]. After DdCBEs, another type of mitochondrial base editor was developed, named TALE-linked deaminases (TALEDs), which mediate A-T to G-C transitions in mtDNA [44]. The development of mitochondrial base editors has led to the identification of potential therapeutic targets for treating HSC disease caused by mtDNA mutations.

## Advances in Base Editor-Mediated Hematopoietic Stem Cell Gene Therapy

Due to the multidirectional differentiation potential of HSCs, their gene editing allows for durable gene correction in different lineages [45]. The self-renewal ability allows for permanent transmission of gene modifications to progeny cells through precise genome editing of HSCs [46]. Thus, HSCs are ideal targets for gene therapy. Moreover, DSB-free base editors are more precise and safer than other gene editing tools, enabling effective genome editing of HSCs. Therefore, base editor-mediated gene therapy in HSCs can be used to treat a variety of diseases, mainly including hemoglobinopathies and immunodeficiency diseases.

### Ex Vivo

The main hemoglobinopathies are SCD and β-thalassemia. SCD is an autosomal recessive hemolytic disease characterized by the sickling of erythrocytes at low oxygen concentrations. The pathogenesis of this disease is based on an A > T mutation in the hemoglobin subunit beta (*HBB*) gene that generates the β^S^ allele, resulting in the replacement of glutamic acid with valine at position 6 of the mature β-globin chain. Individuals with only one β^S^ allele have no obvious clinical manifestations, and those with two β^S^ alleles (β^S^/β^S^) exhibit SCD [47]. The main clinical manifestations of SCD are jaundice, anemia, and hepatosplenomegaly [48].

**Fig. 3:**
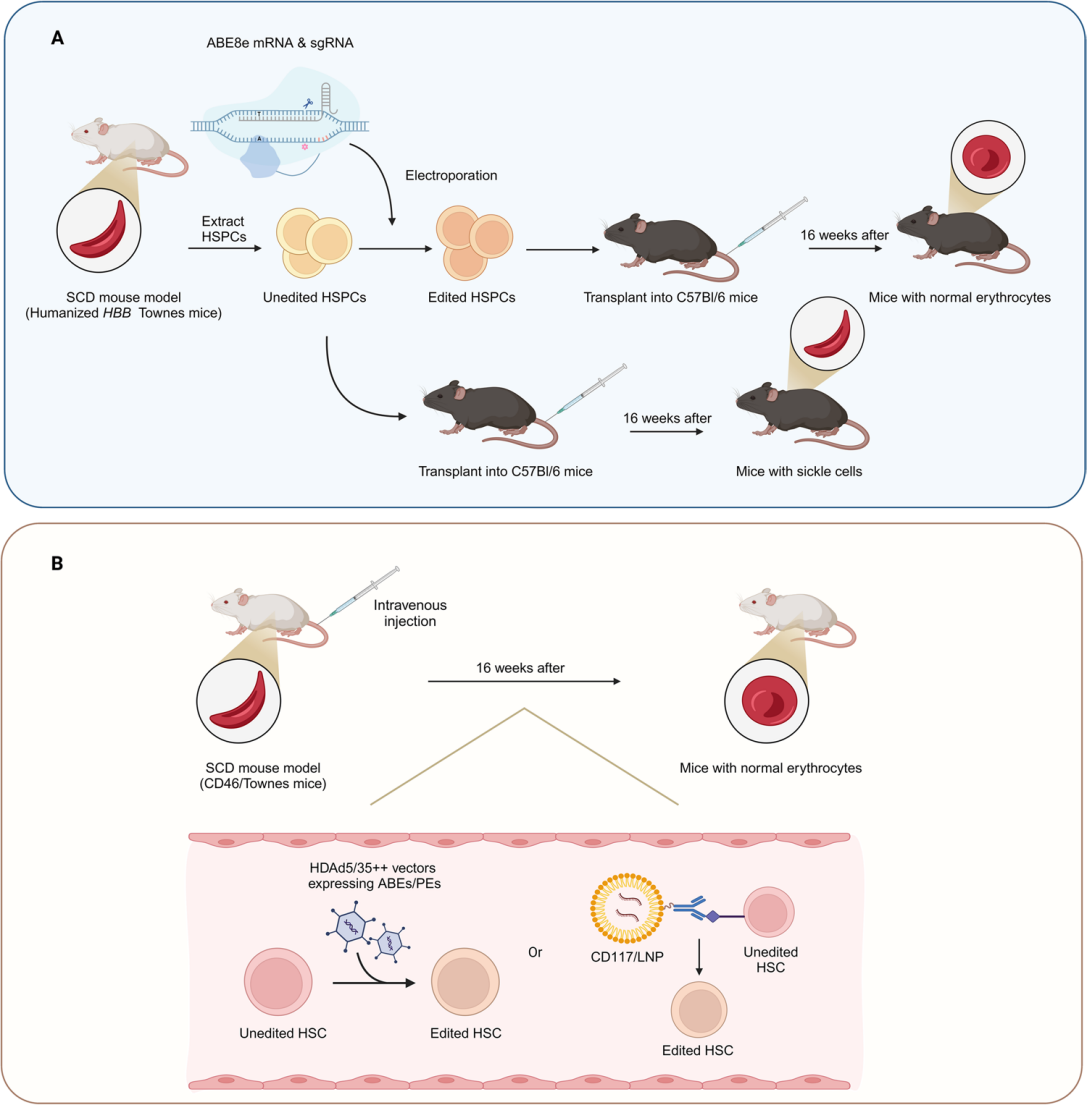
Experimental base editor-based gene therapy for hematopoietic stem cells. **a** Ex vivo base editing. First, HSPCs from the SCD mouse model were extracted, and ABE8e mRNA and sgRNA were subsequently introduced into unedited HSPCs by electroporation. Second, the edited HSPCs and the unedited HSPCs were transplanted into two groups of C57BL/6 mice. Finally, after 16 weeks, most of the erythrocytes in the mice transplanted with the edited HSPCs returned to normal, while most of the erythrocytes in the mice transplanted with the unedited HSPCs were sickle cells. **b** In vivo base editing. The vector delivering the base editor was introduced into the SCD mouse model by intravenous injection. The delivery routes included transfection of HSCs with HDAd5/35^++^ vectors and targeting of LNPs to CD117 on the surface of HSCs with an anti-CD117 antibody. A preponderance of normal erythrocytes was observed in blood extracted after 16 weeks. Created with BioRender.com

Currently, there are limited treatment options for SCD. In previous studies, clinical trials have been conducted using the CRISPR/Cas9 system to treat patients. However, gene therapy strategies based on the CRISPR/Cas9 system have led to several adverse events [49]. It has been reported that repair of double-strand breaks induced by CRISPR/Cas9 leads to large deletions and complex rearrangements [50]. Furthermore, CRISPR/Cas9 globin editing can also induce megabase-scale copy-neutral losses of heterozygosity in hematopoietic cells [51]. Given the many adverse effects caused by DSBs, selecting DSB-free base editors for HSC gene therapy can minimize the above adverse events. Newby et al. [52] used ABE8e-NRCH to repair pathogenic point mutations in HSPCs from SCD patients ex vivo. ABE8e-NRCH converted the SCD β-globin gene (*HBB*^*S*^) into the nonpathogenic Makassar β-globin gene (*HBB*^*G*^) (Fig. 3a). Pathogenic β^S^ proteins were reduced 5.1-fold in edited cells compared to unedited cells. After the researchers delivered ABE8e-NRCH ribonucleoprotein complexes (RNPs) via electroporation into HBB^S/S^ HSPCs from SCD mice and transplanted them into mice, the SCD mice had an average of 75–82% total β-globin, threefold reduction in hyperoxia-induced sickle cells and a return to near-normal spleen size. Recently, Everette et al. [53] used PE3max to edit HSPCs from SCD patients ex vivo, with HBB^S^ editing efficiencies ranging from 15–41%. Sixteen weeks after the edited HSPCs were transplanted into immunodeficient mice, an average of 42% of human erythrocytes and reticulocytes expressed HBB^A^, which improved anemia symptoms.

Beta-thalassemia is an autosomal recessive blood disorder characterized by reduced synthesis (β^+^) or deletion (β^0^) of the β-globin chain of the hemoglobin tetramer and is classified into three types: thalassemia minor, thalassemia intermedia, and thalassemia major [54]. Among them, thalassemia minor has no obvious clinical symptoms, and thalassemia major clinically manifests as growth retardation, pallor, jaundice, hepatosplenomegaly, and skeletal deformity [55].

Direct correction of pathogenic point mutations using base editors to restore β-globin could be utilized to treat β-thalassemia. IVS1-110 (G > A) is one of the most common mutations in patients from the Middle East and the Mediterranean region. Hardouin et al. [56] electroporated sgRNAs into HSPCs of patients with β-thalassemia with SpRY-ABE8e mRNA to correct the G > A mutation. The treated erythrocytes expressed higher levels of β-globin. Researchers further transplanted edited HSPCs into immunodeficient mice, and the expression of β-globin and the α-globin/non-α-globin ratio were effectively restored in the corrected group of mice compared to those in the group of mice transplanted with non-SpRY-ABE8e-edited HSPCs.

Fetal hemoglobin (HbF), which consists of γ-globin and α-globin, is found at high levels in newborns. Adults predominantly express adult hemoglobin (HbA) with minimal amounts of HbF [57]. Gene therapy strategies for SCD and β-thalassemia are based on increasing HbF levels to compensate for the defects in HbA levels, which is a universal approach for treating hemoglobinopathies [58–60]. Zeng et al. [60] electroporated A3A (N57Q)-BE3, which targets the + 58 erythroid BCL11 transcription factor A (*BCL11A*) enhancer with sgRNA, into human CD34^+^ HSPCs and induced production of HbF through base editing to disrupt the erythroid BCL11A enhancer. Researchers edited CD34^+^ HSPCs from two β-thalassemia patients with editing efficiencies of 93.3% and 90.6% and observed an increase in erythrocyte size and a rounder erythrocyte shape. Recently, Mayuranathan et al. [61] introduced an A-T to G-C mutation at a position -175 bp upstream of the γ-globin transcriptional start site via ABE8e, which effectively induced the production of HbF and resulted in a significant reduction in the number of hypoxia-induced sickle cells. Base editing of -175A > G was more potent at inducing HbF than the use of Cas9 to disrupt the + 58 BCL11A erythroid enhancer or the γ-globin promoter BCL11A binding motif.

Other than hemoglobinopathies, base editing gene therapy of HSCs/HSPCs is not widely used at present, with only a few exceptions. Fanconi anemia (FA) is a rare autosomal or X-linked recessive disorder characterized by aplastic anemia, cancer susceptibility and developmental abnormalities. The distinguishing feature of FA is the impairment of the body's ability to maintain genome integrity, which results in the accelerated accumulation of key genetic changes that promote cellular transformation and increase the chances of cancer. Mutations in any of the genes in the FA-BRCA pathway, including FA complementation group A (*FANCA*), *FANCC*, and *FANCG*, may cause FA [62]. Siegner et al. [63] used ABE8e to edit CD34^+^ HSPCs ex vivo from three FA patients harboring the *FANCA* c.295 C > T mutation. Five days after electroporation, the editing efficiency was 57.51 ± 21.00%, 64.37%, and 42.22% for the three patients. This result demonstrated the high efficiency of ABE8e for editing HSPCs from FA patients. However, due to the extreme scarcity of HSPCs from FA patients, the investigators did not transplant ABE8e-edited cells into immunodeficient mice to verify whether ex vivo gene editing was effective. We expect relevant trials to fill this gap in the future.

CD3δ severe combined immune deficiency (SCID) is an autosomal disorder caused by a point mutation (c.202C > T) of the CD3 delta subunit of the T-cell receptor complex (*CD3D*). Patients present with a decrease in αβ T cells and γδ T cells, often resulting in infant mortality [64]. McAuley et al. [65] first precisely restored the *CD3D* c.202C > T mutation in HSPCs from CD3δ SCID patients ex vivo using ABEmax-NRTH, with a correction rate of 71.2% ± 7.85% for the pathogenic mutation. Next, to investigate the ability of ABEs to correct mutations in the long term in HSPCs from CD3δ SCID patients, the investigators electroporated mRNA encoding ABEmax-NRTH and sgRNA into human CD34^+^ HSPCs from healthy donors transduced with lentiviral vectors carrying a *CD3D* c.202C > T mutation and transplanted them into immunodeficient mice. Sixteen weeks after transplantation, the mice showed durable editing efficiency throughout the bone marrow, spleen, and thymus, and no alterations in hematopoiesis were detected. Finally, to investigate whether ABE-edited CD3δ SCID HSPCs could be used to restore the normal function of T cells, the authors used 3D artificial thymic organoids (ATOs) to determine the surface expression of CD3 and the T-cell receptor (TCR) in HSPCs. The results showed that the coexpression of TCR and CD3 on the surface of ATOs from ABE-edited patients was significantly increased in double-positive (DP, CD4 + CD8 +) T cells, single-positive eight (SP8, CD3 + TCRαβ + CD4-CD8α + CD8β +) T cells and single-positive four (SP4, CD3 + TCRαβ + CD4 + CD8α-) T cells. Taken together, these findings suggest that ABE-mediated gene therapy restores T-cell development in patients with CD3δ SCID, providing a treatment approach for this disease.

### In Vivo

The above experimental results provide a promising strategy for the treatment of sickle cell disease, β-thalassemia, and other nonmalignant hematopoietic diseases utilizing base editors to modify autologous HSCs ex vivo and subsequently transplanting these cells into the body to restore defective hemoglobin expression. However, this autologous stem cell therapy is not only costly but also more painful for patients, which may cause systemic damage such as infections and cancer. Compared with ex vivo editing, in vivo editing of HSCs is technically much simpler, as the drug can be delivered by simple intravenous infusion, and there is no need for pretransplantation conditioning that can lead to toxicity. Therefore, this approach could become a major trend for the treatment of nonmalignant hematopoietic diseases in the future.

Before discussing the therapeutic opportunities of base editors, it is necessary to briefly summarize the in vivo delivery strategies of base editors, which are critical for therapeutic efficacy. Delivery vectors need to be appropriately sized to encapsulate the base editor DNA or mRNA, and they need to have the appropriate molecules on their surface to bind to the receptor on the surface of the HSCs [66]. In general, we can categorize delivery vectors into viral and nonviral vectors. There are three main types of viral vectors: adenoviral vectors, lentiviral vectors, and adeno-associated viral vectors, among which the most widely used in HSCs are adenoviral vectors. Adenoviruses are particles with a diameter of 90–100 nm without an envelope and consist of 252 capsids arranged in a 20-sided configuration. The genome of the commonly-used human adenovirus type 5 (Ad5) is approximately 36 kb [67]. Helper-dependent adenovirus (HDAd) vectors lack the viral coding region, allowing them to elicit little cellular immune response. Additionally, they have a large cloning capacity, making them suitable as efficient delivery vectors [68]. A widely used system for delivering base editors into HSCs is the HDAd5/35^++^ gene transfer vector system established by Lieber's team, which can efficiently target CD46 on the surface of HSPCs. The advantages of this vector include high capacity, low production cost, and lack of significant cytotoxicity [69]. Recently, the use of lipid nanoparticles (LNPs) to deliver base editors has been reported to directly modify hematopoietic stem cells in vivo [70]. Currently, LNPs are not commonly used for in vivo HSC editing compared to viral vectors. As a promising delivery vector, it leads to transient expression of base editors, which reduces off-target editing. However, LNPs are coated by ApoE lipoproteins after injection into the blood, which leads to uptake by the liver. Therefore, it is often used for gene editing in the liver, but for this very reason it does not target HSCs precisely, which may reduce in vivo therapeutic efficacy [66].

Li et al. [71] developed an in vivo base editing therapy based on helper-dependent adenovirus 5/35^++^ (HDAd5/35^++^) vectors that target CD46 expressed on HSPCs. The researchers used ABEmax to reconstruct the hereditary persistence of fetal hemoglobin (HPFH) mutations in the hemoglobin subunit gamma 1/2 (*HBG1/2*) promoter (Fig. 3b). Sixteen weeks after vector injection, the level of γ-globin in the peripheral blood erythrocytes of the mice increased from 1 to 43%. Similarly, the research team utilized the developed HDAd-EF1α vector to deliver ABE8e into β-YAC/CD46 mice by intravenous injection. The ABE8e vector targets CD46 expressed on primitive HSCs. Using ABE8e to install the –113 A > G HPFH mutation in the *HBG1/2* promoter, researchers achieved an in vivo editing efficiency of more than 60%, and the γ-globin content in peripheral blood erythrocytes was significantly increased [72]. The research team also used HDAd5/35^++^ vectors to deliver PEs via intravenous injection into an SCD mouse model to repair HBB^S^ mutations. The in vivo editing efficiency in mice averaged 43.6% after 16 weeks of intravenous injection. Compared with untreated mice, 43% of sickle hemoglobin (HbS) was restored to HbA, and the percentage of sickle cells decreased from 86% to 29.6% in blood samples collected from in vivo lead-edited mice. Moreover, the spleen size of the edited mice was significantly reduced [73].

Breda et al. [70] constructed an LNP that targets HSCs; the CD117 antibody on the surface of the LNP binds to the CD117 antigen on HSCs, allowing the LNP to accurately deliver ABE mRNA and sgRNA into HSCs. This method enables in vivo modification of HSCs through simple intravenous injection with fewer side effects, providing new possibilities for the treatment of hereditary blood disorders. With continuous advancements in technology and in-depth research, in vivo editing as HSC therapy is expected to be further promoted and applied in the treatment of more diseases (Table 2).

**Table 2:** Advances in base editing technology-mediated hematopoietic stem cell gene therapy

Disease	Edited cell type	Delivery system	Base/Prime editor	Target gene	References
Ex vivo					
Sickle cell disease	HSPCs	RNP	ABE8e-NRCH	HBB	[52]
Sickle cell disease		mRNA	PE3max	HBB	[53]
Beta-thalassemia		mRNA	SpRY-ABE8e	HBB	[56]
Sickle cell disease/Beta-thalassemia		RNP	A3A (N57Q)-BE3	BCL11A erythroid enhancer	[60]
Sickle cell disease/Beta-thalassemia		LNP	ABE8e	HBG1/2 promoter	[61]
Fanconi anemia		mRNA	ABE8e	FANCA	[63]
CD3δ SCID		mRNA	ABEmax-NRTH	CD3D	[65]
In vivo					
Sickle cell disease/Beta-thalassemia	HSPCs	Ad	ABEmax	HBG1/2 promoter	[71]
Sickle cell disease/Beta-thalassemia	HSCs	Ad	ABE8e	HBG1/2 promoter	[72]
Sickle cell disease		Ad	PE5max	HBB	[73]
Sickle cell disease		LNP	ABE8e	HBB	[70]

However, in vivo editing of HSCs still has shortcomings, such as the inflammatory reaction triggered during intravenous injection. Additionally, the long-term presence of adenoviral vectors in the body may increase the risk of cancer [74]. Another more serious problem is that since the CD46 receptor for HDAd5/35^++^ is expressed on all nucleated cells, viral vectors have the potential to be transduced into nonhematopoietic tissues; for example, vector genomes have been detected in the liver and lungs [73]. This off-target transduction may increase the probability of undesired genetic modifications and be harmful to the patient’s health. Therefore, finding receptors that are more specifically expressed on HSCs, such as stem cell factor receptors, is key to improving editing efficiency and enhancing the specificity of in vivo editing.

## Prospects of Base Editor-Mediated Hematopoietic Stem Cell Gene Therapy for Different Inherited Hematologic Diseases

### Erythrocyte Disease

α-Thalassemia is an autosomal recessive disorder manifested by defective production of α-globin in HbA (α_2_β_2_). α-Globin is regulated by two α-globin genes on each of a pair of chromosome 16, and the genotype of normal individuals is αα/αα. Depending on the type of mutation, the disease can be divided into deletional α-thalassemia and nondeletional α-thalassemia. α^0^-Thalassemia refers to the complete absence of chromosomal expression of the α gene, whereas α^+^-thalassemia refers to the downregulation of chromosomal α gene expression. Nondeletional α^+^-thalassemia tends to be more severe than deletional α-thalassemia [75].

Like the induction of γ-globin production in the treatment of β-thalassemia and SCD, the activation of embryonically expressed ζ-globin likewise compensates for the lack of α-globin. King et al. [76] reported that the δ-gene interacts with the α-globin super-enhancer in embryonic erythroid cells and is located inside an ~ 65 kb subtopologically associating domain (sub-TAD) of open, acetylated chromatin. However, in adult erythroid cells, the ζ-gene is contained within an approximately 10 kb subdomain of hypoacetylated facultative heterochromatin within the acetylated sub-TAD, and it is no longer in contact with its enhancers [76]. Hence, reactivation of the ζ-gene in HSCs could be a potential way to treat α-thalassemia. Besides, Li et al. [77] used the CRISPR/Cas9 system to repair the Hb WS mutation in the human hemoglobin alpha 2 (*HBA2*) gene. The edited cells were human induced pluripotent stem cells (hiPSCs) derived from the patient's amniotic cells and could be differentiated into hematopoietic progenitor cells ex vivo [77]. In the same way, the nondeletional types of α-thalassemia caused by single gene mutations, such as Hb Constant Spring (Hb CS, c.427 T > C), Hb Quong Sze (Hb QS, c.377 T > C) and Hb Westmead (Hb WS, c.369 C > G), could be theoretically corrected by base editors in HSCs.

Diamond–Blackfan anemia (DBA) is an autosomal dominant form of erythrocyte hypoplasia caused mainly by pathogenic germline variants of ribosomal protein genes, such as ribosomal protein S19 (*RPS19*), *RPS24*, *RPS17*, *RPL5*, *RPL11* and *RPL35A*, with clinical manifestations such as severe anemia, skeletal malformations, and cancer predisposition [78]. Recently, it has been shown that biallelic variants in HEAT repeat containing 3 (*HEATR3*) lead to impairment of nuclear import of uL18 (RPL5) and erythropoiesis, resulting in DBA [79]. Current treatment for DBA is based on corticosteroid therapy, chronic red blood cell infusion, or HSCT [80]. HSC-based base editing therapies could be alternative strategies for the treatment of DBA in the future.

### Primary Immunodeficiency Disease

Wiskott–Aldrich syndrome (WAS) is an X-linked recessive immunodeficiency disorder characterized by thrombocytopenia, eczema, recurrent infections, and an increased risk of autoimmune diseases and malignancies. Mutations in the WASP actin nucleation promoting factor (*WAS*) gene result in defective synthesis of the WAS protein (WASp), a protein expressed in nearly all hematopoietic cells that is involved in the polymerization of the actin skeleton; decreased levels of this protein result in defective immune cell function. The current effective treatment is HSCT [81]. There are many pathogenic *WAS* mutation sites; Jin et al. [82] identified and characterized a total of 141 unique *WAS* mutations, the most common of which were missense mutations. Rai et al. [83] developed a CRISPR/Cas9-based gene therapy strategy to allow transcriptional regulation of WAS-regulated regions by knocking in therapeutic *WAS* cDNA with endogenous translation initiation codons in patient HSPCs. This is a universal strategy for all mutations. However, CRISPR/Cas9 is more genotoxic than base editors for ex vivo editing of HSPCs (discussed previously), potentially leading to some adverse effects. Among all the *WAS* mutations, c.168C > T, c.290C > N/291G > N, and c.665C > T are the most common point mutations and can be used as target sites for in situ repair by base editors [82].

SCIDs are a group of rare congenital disorders characterized by impaired humoral and cellular immunity, leukopenia, and low or absent antibody levels [84]. Previously, base editing against CD3δ SCID was reported. However, the most common type is X-linked SCID caused by mutations in the interleukin 2 receptor subunit gamma (*IL2RG*) gene. Among all the *IL2RG* point mutations, c.690C > T, c.691G > A, c.684C > T, c.879C > T, and c.868G > A are the most common and therefore suitable for base editing [85]. As for WAS, the use of CRISPR/Cas9 to edit HSCs for the treatment of X-linked SCID has been reported [86].

X-linked agammaglobulinemia (XLA) is another primary immunodeficiency disease caused by mutations in the Bruton tyrosine kinase (*BTK*) gene. Mutations in the *BTK* gene result in defective synthesis of Bruton tyrosine kinase (BTK), which leads to disruption of the maturation of pre-B cells into B cells [87]. Currently, the first-line treatment for XLA is intravenous gammaglobulin replacement therapy (IVIG) and HSCT. In China, the most common recurrent point mutations in *BTK* are in arginine-coding CpG dinucleotides, such as c.1559 G > A. Therefore, related point mutations could be sites for in situ repair by base editors [88].

### Metabolic Diseases

Unlike the diseases mentioned above, Gaucher disease (GD) is a metabolic disease, for which gene therapy can also be achieved by editing HSPCs. GD, caused by mutations in the glucosylceramidase beta 1 (*GBA1*) gene, is a rare autosomal recessive genetic disease. Glycolipids accumulate in macrophages due to glucocerebrosidase (GCase) deficiency, resulting in clinical symptoms such as hepatosplenomegaly, anemia, and bone disease [89]. In a previous study, Scharenberg et al. [90] utilized the CRISPR/Cas9 and adeno-associated virus (AAV) systems to target GCase expression cassettes to the human CCR5 safe harbor locus in HSPCs, realizing GCase expression in monocyte/macrophage lineages. Of all the *GBA*1 point mutations, the N370S mutation (c.1226 A > G) is the most prevalent; therefore, it is a potential site for in situ repair by base editors [91] (Table 3).

**Table 3:** Prospects of base editors-mediated hematopoietic stem cell gene therapy

Disease	Target gene	Common point mutation	Alternative base editors	Target cell type	References
Erythrocyte disease					
Alpha-thalassemia	*HBA2*	c.427 T > C	CBEs	Erythrocyte	[75–77]
Diamond–Blackfan anemia	*RPS19, RPS24, RPS17, RPL5, RPL11, RPL35A, HEATR3*	c.418 G > A (*RPL5*)	ABEs	Erythrocyte	[78–80]
Primary immunodeficiency disease					
Wiskott–Aldrich syndrome	*WAS*	c.168 C > T	ABEs	Platelet/Neutrophil	[81–83]
X-linked SCID	*IL2RG*	c.690 C > T	ABEs	T/B/NK cell	[85, 86]
X-linked agammaglobulinemia	*BTK*	c.1559 G > A	ABEs	B cell	[87, 88]
Metabolic disease					
Gaucher disease	*GBA1*	c.1226 A > G	CBEs	Macrophage	[89–91]

## Off-Target Editing by Base Editors

Off-target editing of base editors is a major obstacle limiting their clinical application. Off-target editing induced by base editors can be classified into three main categories: Cas-dependent off-target editing, Cas-independent off-target editing, and RNA off-target editing. CBEs and ABEs can recognize a small number of off-target sites and perform C-G to T-A or A-T to G-C off-target edits within the active window. These off-target sites have similar sequences to those of sgRNAs, and editing at these sites is caused by the degree of nonspecificity of the Cas9 nuclease, which allows it to tolerate base mismatches; this process is referred to as Cas-dependent off-target editing [25, 92, 93]. Recently, the mismatches tolerated by the Cas9 nuclease were revealed to be achieved through the formation of noncanonical base pairings [94].

To avoid Cas9-dependent off-target editing, researchers have developed a series of high-fidelity Cas9 nucleases and constructed corresponding base editors. Rees et al. [95] introduced four point mutations in the high-fidelity Cas9 variant HF-Cas9 into BE3, constructing HF-BE3, which was designed to eliminate nonspecific interactions between Cas9 and DNA. At the highly repetitive VEGFA2 site, HF-BE3 exhibited a threefold reduction in off-target editing efficiency [95]. Lee et al. [96] established a directed evolution model in *E. coli*, screened it to obtain Sniper-Cas9 with high specificity for target sequences, and constructed Sniper-BE3, which showed a 2.4- to 16.2-fold greater editing efficiency at specific off-target sites than did a wild-type Cas9-constructed BE3. Furthermore, researchers have shown that delivery via RNPs can reduce the duration of the base editor's action in the cell compared to delivery via plasmids, similarly reducing Cas-dependent off-target editing [96].

In addition to the nonspecificity of Cas9 leading to off-target editing, the nonspecificity of deaminases can also lead to off-target editing and is more common in CBEs. Zuo et al. [97] used self-developed genome-wide off-target analysis via two-cell embryo injection (GOTI) off-targeting monitoring technology and observed that the number of SNVs induced by BE3 in mouse embryos was much greater than that induced by ABE or CRISPR/Cas9, and this off-target editing was difficult to predict using existing off-target prediction methods. Jin et al. [98] also observed similar results in rice. These SNVs were predominantly C-G to T-A, and the regions containing the SNVs were not similar to the sgRNA sequences, suggesting that these off-target edits are independent of the Cas9 nuclease and are caused by random deamination; therefore, they are referred to as Cas-independent off-targets [98].

To avoid Cas-independent off-target editing, researchers have optimized deaminases to alter their deamination activity. Doman et al. [99] introduced point mutations to deaminases and constructed CBEs, including R33A + K34A-BE4 and R33A + K34A + H122L + D124N-BE4 (known as AALN-BE4), which reduced the Cas-independent off-target editing to less than 0.4%. Yu et al. [100] developed eight next-generation CBEs with low off-target activity that exhibited approximate editing efficiencies and an overall reduction of up to 45-fold in Cas-independent off-target editing compared to those of BE4. Yuan et al. engineered the cytosine deaminase APOBEC3A to generate eA3A-RL1 by introducing the N57G point mutation as well as by introducing the recognition loop region RL1 of APOBEC3G in place of the original RL1. Researchers subsequently utilized N-terminal fusion strategies to generate the above deaminase mutant N-eA3A-RL1-BE, which demonstrated robust editing activity with minimized Cas-independent off-target edits [101]. Additionally, Zhang et al. [102] developed a series of miniCBEs by fusing the reprogrammed deaminase TadA-8e with Cas12f, resulting in the elimination of Cas-independent off-target effects that is comparable to Doman et al.'s report [99].

In addition to these two types of off-target editing, CBEs or ABEs can also cause RNA deamination at the transcriptional level in human cells, leading to RNA off-target editing [103]. To optimize base editors that can selectively avoid RNA editing, Grünewald et al. [104] screened two rAPOBEC1 variants and used them to construct BE3 (R33A) and BE3 (R33A/K34A), which were named SECURE-BE3. Next, the researchers constructed miniABEmax (K20A/R21A) with mini ABEmax (V82G), referred to as SECURE-ABEs [104]. All these variants significantly reduced the amount of RNA editing in human cells.

The genotoxic effects of ex vivo base editing in HSCs cannot be ignored, which is closely related to the off-target editing effect of base editors. Fiumara et al. [105] targeted the beta-2-microglobulin (*B2M*) gene by using BE4max and ABE8.20-m to test their knockout effect on HSCs. Sequencing revealed that BE4max editing produced indels at the target site in more than 1/3 of the alleles. BE4max also triggered p53 pathway activation, and all the base editors in this study also activated interferon alpha (IFNα) and IFNγ responses. Moreover, researchers have shown that BE4max impairs the long-term engraftment of edited HSPCs, a phenomenon not observed with ABEs [105]. This difference may be due to the greater amount of off-target editing caused by CBEs. Yan et al. [106] observed an abnormal phenotype of obesity and developmental delay in mice with permanent overexpression of the BE3 gene during long-term monitoring, and two developmentally delayed dead mice overexpressing the BE3 gene carried a fivefold greater number of DNA mutations compared to the control group. Thus, it is particularly important to ensure that the off-target editing of base editors is minimized. The establishment of more accurate off-target detection methods and further development of high-fidelity base editors will help to push base editing technology into clinical applications more quickly. In addition to the genotoxicity of base editors, the body's innate immune system is another major obstacle. Ex vivo modification of the HSC genome may lead to the expression of neoantigens, which may trigger an immune response [74].

## Improving the Editing Efficacy of Base Editors in HSCs

### Selecting Appropriate Base Editors

In all the experiments utilizing base editors to modify HSCs, the majority of the base editors were ABEs (Table 4). This result is consistent with previous reports that CBEs produce a large number of off-target edits as well as genotoxicity in HSCs [97, 105]. Therefore, we suggest that it is better to choose ABEs rather than CBEs for editing HSCs to avoid the adverse effects associated with off-target editing. However, recent advances, such as TadCBEs and tBEs, have substantially reduced Cas-independent off-target editing compared to other CBEs and thus may hold promise in HSCs [28, 107].

**Table 4:** The three most common base editors for therapeutic gene editing in HSCs

Base editor	Architecture	Properties	References
A3A(N57Q)-BE3		Preferential deamination of cytosine in TCR motif to minimize bystander editing	[60]
ABEmax		Moderate editing efficiency, fewer off-target editing, fewer bystander editing	[65, 71]
ABE8e		Robust editing efficiency, more off-target editing, more bystander editing	[52, 61, 63, 70, 72]

In addition to selecting base editors with low off-target activity, the following points should be noted when selecting base editors for HSC gene editing: 1. PAM restriction. Most BEs currently use the SpCas9 nuclease, which recognizes the NGG PAM [18]. However, this may limit the editing scope of base editors. Therefore, base editors that are not restricted by PAM sequences can be selected. For example, SpRY-ABE has been reported to be effective at repairing the β-thalassemia IVS1-110 (G > A) mutation [56, 108]. 2. Product purity. A small number of indels and byproducts can be induced in the base editor, which may impair the effectiveness of HSC editing. For example, high-frequency indels in the BCL11A erythroid enhancer disrupt functional GATA motifs, thereby preventing efficient disruption of enhancer activity [61]. To avoid this disadvantage, base editors that reduce the frequency of indels such as BE4-GAM, can be selected [24]. 3. Bystander editing. When bystander editing is unavoidable and may have adverse results, base editors with narrow activity windows or with contextual preference should be selected to avoid bystander editing. For example, the A3A (N57Q)-BE3 chosen by Zeng et al. [60]. This base editor prefers to deaminate cytosine in TCR motifs, resulting in potent HbF induction in vivo [109]. However, the occurrence of bystander editing in HSCs may not always be detrimental. Bystander editing might sometimes promote in vivo editing efficiency. For instance, ABE promotes HbF activation by achieving the -113A > G transition to disrupt BCL11A within the HBG1/2 promoter. In this process, bystander editing at the -116 site facilitates the disruption of BCL11A, thereby further promoting HbF activation [72]. In this case, a base editor with an appropriately enlarged activity window can be selected to maximize the editing efficiency.

### Minimizing Genetic Heterogeneity

Gene editing tools, especially CRISPR/Cas9 that yields DSBs, usually result in the generation of genetically heterogeneous populations of HSCs [110]. Compared to CRISPR/Cas9, DSB-free base editors produce less heterogeneous populations, but this still cannot be ignored. In brief, genetic heterogeneity caused by base editors results from three main sources: off-target editing, byproducts, and indels. This could be potentially harmful in gene therapy. Therefore, to minimize the effect of genetic heterogeneity in the population of HSCs, the following measures are available. 1. Select base editors with low off-target activity, fewer byproducts, and decreased indel frequencies (discussed previously). It is worth noting that all three of these disadvantages are more pronounced in CBEs rather than in ABEs [26]. 2. Optimize ex vivo clone culture systems. Recently, Becker et al. developed a strategy for single HSC expansion by replacing PVA with Soluplus, an amphiphilic polyvinyl caprolactam-acetate polyethylene glycol (PCL-PVAc-PEG) graft copolymer. Researchers have estimated that a single HSC expansion using Soluplus can be amplified more than 33,000-fold, and no nonsynonymous mutations were found in key genes in the expanded HSC clones. The effectiveness of this ex vivo expansion platform was demonstrated in the *Prkdc*^*scid*^ immunodeficiency model, which offers the prospect of ex vivo gene therapy for HSCs [110].

## Conclusions

Base-editing therapies based on HSCs have brought new hope to patients with hematologic diseases because the precision of these treatments starts at the root of the gene mutations. This new therapy builds on the ongoing development of various base editors. Despite the enormous potential of base editing technology, this emerging therapeutic approach still requires substantial development to move from the laboratory to the clinic. With the development of CGBE, GBE, AYBE, and AXBE, all 12 base substitutions, including transitions and transversions, have been realized. In other words, base editors can achieve substitutions between any two bases [12, 13, 39, 111]. However, for transversion editors, realizing two types of base substitutions at the same time reduces their selectivity and clinical application value. Compared with base editors, PEs can perform all 12 base substitutions as well as more diverse gene editing; however, their editing efficiency may not be as high as that of base editors [14]. Furthermore, the accuracy and efficiency of base editing can be improved, and the design of editing systems can be optimized by deep learning, machine learning, and other artificial intelligence technologies [112]. The choice of base editor is crucial for the characterization of different mutations in different diseases. Currently, several deep learning-based base editing methods, as well as off-target prediction websites, have been developed, and researchers can refer to the base editing efficiency and off-target efficiency predicted by these websites to select the appropriate base editors, which will effectively improve the efficiency of the experiments [113–115]. Currently, base editing therapies targeting HSCs are entering the clinical trial stage. For example, Beam-101, developed by Beam Therapeutics, treats SCD and β-thalassemia by promoting the expression of HbF and has entered phase I clinical trials [4]. Although these base-editing therapies have shown potential efficacy, their safety needs to be further investigated and validated, as they are still in the early stages of research. As researchers increasingly emphasize safety and bioethics in the field of gene therapy, we expect an increasing number of base editing technology-mediated HSC gene therapies to lead to breakthroughs in the future.

## Data Availability

Not applicable.

## References

[1] Copelan, E. A. (2006). Hematopoietic stem-cell transplantation. *The New England Journal of Medicine,**354*(17), 1813–1826. 10.1056/NEJMra052638 16641398 10.1056/NEJMra052638

[2] Boelens, J. J., Aldenhoven, M., Purtill, D., Ruggeri, A., Defor, T., Wynn, R., Wraith, E., Cavazzana-Calvo, M., Rovelli, A., Fischer, A., Tolar, J., Prasad, V. K., Escolar, M., Gluckman, E., O’Meara, A., Orchard, P. J., Veys, P., Eapen, M., Kurtzberg, J., … Eurocord, Blood, I. E. W. P. o. E., Marrow Transplant Group, Duke University Blood and Marrow Transplantation Program, Blood, C. f. I., & Marrow Research. (2013). Outcomes of transplantation using various hematopoietic cell sources in children with Hurler syndrome after myeloablative conditioning. *Blood,**121*(19), 3981–3987. 10.1182/blood-2012-09-455238 23493783 10.1182/blood-2012-09-455238 PMC3836041

[3] Cooke, K. R., Luznik, L., Sarantopoulos, S., Hakim, F. T., Jagasia, M., Fowler, D. H., Van Den Brink, M. R. M., Hansen, J. A., Parkman, R., Miklos, D. B., Martin, P. J., Paczesny, S., Vogelsang, G., Pavletic, S., Ritz, J., Schultz, K. R., & Blazar, B. R. (2017). The biology of chronic graft-versus-host disease: A task force report from the national institutes of health consensus development project on criteria for clinical trials in chronic graft-versus-host disease. *Biology of Blood and Marrow Transplantation,**23*(2), 211–234. 10.1016/j.bbmt.2016.09.023 27713092 10.1016/j.bbmt.2016.09.023 PMC6020045

[4] Kingwell, K. (2022). Base editors hit the clinic. *Nature Reviews Drug Discovery,**21*(8), 545–547. 10.1038/d41573-022-00124-z 35831515 10.1038/d41573-022-00124-z

[5] Maeder, M. L., & Gersbach, C. A. (2016). Genome-editing technologies for gene and cell therapy. *Molecular Therapy,**24*(3), 430–446. 10.1038/mt.2016.10 26755333 10.1038/mt.2016.10 PMC4786923

[6] Jinek, M., Chylinski, K., Fonfara, I., Hauer, M., Doudna, J. A., & Charpentier, E. (2012). A programmable dual-RNA-guided DNA endonuclease in adaptive bacterial immunity. *Science,**337*(6096), 816–821. 10.1126/science.1225829 22745249 10.1126/science.1225829 PMC6286148

[7] Takata, M., Sasaki, M. S., Sonoda, E., Morrison, C., Hashimoto, M., Utsumi, H., Yamaguchi-Iwai, Y., Shinohara, A., & Takeda, S. (1998). Homologous recombination and non-homologous end-joining pathways of DNA double-strand break repair have overlapping roles in the maintenance of chromosomal integrity in vertebrate cells. *The EMBO Journal,**17*(18), 5497–5508. 10.1093/emboj/17.18.5497 9736627 10.1093/emboj/17.18.5497 PMC1170875

[8] Cox, D. B., Platt, R. J., & Zhang, F. (2015). Therapeutic genome editing: Prospects and challenges. *Nature Medicine,**21*(2), 121–131. 10.1038/nm.3793 25654603 10.1038/nm.3793 PMC4492683

[9] Chapman, J. R., Taylor, M. R., & Boulton, S. J. (2012). Playing the end game: DNA double-strand break repair pathway choice. *Molecular Cell,**47*(4), 497–510. 10.1016/j.molcel.2012.07.029 22920291 10.1016/j.molcel.2012.07.029

[10] Komor, A. C., Kim, Y. B., Packer, M. S., Zuris, J. A., & Liu, D. R. (2016). Programmable editing of a target base in genomic DNA without double-stranded DNA cleavage. *Nature,**533*(7603), 420–424. 10.1038/nature17946 27096365 10.1038/nature17946 PMC4873371

[11] Gaudelli, N. M., Komor, A. C., Rees, H. A., Packer, M. S., Badran, A. H., Bryson, D. I., & Liu, D. R. (2017). Programmable base editing of A*T to G*C in genomic DNA without DNA cleavage. *Nature,**551*(7681), 464–471. 10.1038/nature24644 29160308 10.1038/nature24644 PMC5726555

[12] Zhao, D., Li, J., Li, S., Xin, X., Hu, M., Price, M. A., Rosser, S. J., Bi, C., & Zhang, X. (2021). Glycosylase base editors enable C-to-A and C-to-G base changes. *Nature Biotechnology,**39*(1), 35–40. 10.1038/s41587-020-0592-2 32690970 10.1038/s41587-020-0592-2

[13] Tong, H., Wang, X., Liu, Y., Liu, N., Li, Y., Luo, J., Ma, Q., Wu, D., Li, J., Xu, C., & Yang, H. (2023). Programmable A-to-Y base editing by fusing an adenine base editor with an N-methylpurine DNA glycosylase. *Nature Biotechnology,**41*(8), 1080–1084. 10.1038/s41587-022-01595-6 36624150 10.1038/s41587-022-01595-6

[14] Anzalone, A. V., Randolph, P. B., Davis, J. R., Sousa, A. A., Koblan, L. W., Levy, J. M., Chen, P. J., Wilson, C., Newby, G. A., Raguram, A., & Liu, D. R. (2019). Search-and-replace genome editing without double-strand breaks or donor DNA. *Nature,**576*(7785), 149–157. 10.1038/s41586-019-1711-4 31634902 10.1038/s41586-019-1711-4 PMC6907074

[15] Chen, P. J., & Liu, D. R. (2023). Prime editing for precise and highly versatile genome manipulation. *Nature Reviews Genetics,**24*(3), 161–177. 10.1038/s41576-022-00541-1 36344749 10.1038/s41576-022-00541-1 PMC10989687

[16] Garneau, J. E., Dupuis, M. E., Villion, M., Romero, D. A., Barrangou, R., Boyaval, P., Fremaux, C., Horvath, P., Magadan, A. H., & Moineau, S. (2010). The CRISPR/Cas bacterial immune system cleaves bacteriophage and plasmid DNA. *Nature,**468*(7320), 67–71. 10.1038/nature09523 21048762 10.1038/nature09523

[17] Deltcheva, E., Chylinski, K., Sharma, C. M., Gonzales, K., Chao, Y., Pirzada, Z. A., Eckert, M. R., Vogel, J., & Charpentier, E. (2011). CRISPR RNA maturation by trans-encoded small RNA and host factor RNase III. *Nature,**471*(7340), 602–607. 10.1038/nature09886 21455174 10.1038/nature09886 PMC3070239

[18] Sternberg, S. H., Redding, S., Jinek, M., Greene, E. C., & Doudna, J. A. (2014). DNA interrogation by the CRISPR RNA-guided endonuclease Cas9. *Nature,**507*(7490), 62–67. 10.1038/nature13011 24476820 10.1038/nature13011 PMC4106473

[19] Zetsche, B., Gootenberg, J. S., Abudayyeh, O. O., Slaymaker, I. M., Makarova, K. S., Essletzbichler, P., Volz, S. E., Joung, J., Van Der Oost, J., Regev, A., Koonin, E. V., & Zhang, F. (2015). Cpf1 is a single RNA-guided endonuclease of a class 2 CRISPR-Cas system. *Cell,**163*(3), 759–771. 10.1016/j.cell.2015.09.038 26422227 10.1016/j.cell.2015.09.038 PMC4638220

[20] Makarova, K. S., Wolf, Y. I., Iranzo, J., Shmakov, S. A., Alkhnbashi, O. S., Brouns, S. J. J., Charpentier, E., Cheng, D., Haft, D. H., Horvath, P., Moineau, S., Mojica, F. J. M., Scott, D., Shah, S. A., Siksnys, V., Terns, M. P., Venclovas, Č, White, M. F., Yakunin, A. F., … Koonin, E. V. (2020). Evolutionary classification of CRISPR–Cas systems: A burst of class 2 and derived variants. *Nature Reviews Microbiology,**18*(2), 67–83. 10.1038/s41579-019-0299-x 31857715 10.1038/s41579-019-0299-x PMC8905525

[21] Cong, L., Ran, F. A., Cox, D., Lin, S., Barretto, R., Habib, N., Hsu, P. D., Wu, X., Jiang, W., Marraffini, L. A., & Zhang, F. (2013). Multiplex genome engineering using CRISPR/Cas systems. *Science,**339*(6121), 819–823. 10.1126/science.1231143 23287718 10.1126/science.1231143 PMC3795411

[22] Zhang, X., Wang, J., Cheng, Q., Zheng, X., Zhao, G., & Wang, J. (2017). Multiplex gene regulation by CRISPR-ddCpf1. *Cell Discovery,**3*, 17018. 10.1038/celldisc.2017.18 28607761 10.1038/celldisc.2017.18 PMC5460296

[23] Liu, Y., Han, J., Chen, Z., Wu, H., Dong, H., & Nie, G. (2017). Engineering cell signaling using tunable CRISPR-Cpf1-based transcription factors. *Nature Communications,**8*(1), 2095. 10.1038/s41467-017-02265-x 29235474 10.1038/s41467-017-02265-x PMC5727435

[24] Komor, A. C., Zhao, K. T., Packer, M. S., Gaudelli, N. M., Waterbury, A. L., Koblan, L. W., Kim, Y. B., Badran, A. H., & Liu, D. R. (2017). Improved base excision repair inhibition and bacteriophage Mu Gam protein yields C:G-to-T: A base editors with higher efficiency and product purity. *Science Advances,**3*(8), eaao4774. 10.1126/sciadv.aao4774 28875174 10.1126/sciadv.aao4774 PMC5576876

[25] Porto, E. M., Komor, A. C., Slaymaker, I. M., & Yeo, G. W. (2020). Base editing: Advances and therapeutic opportunities. *Nature Reviews Drug Discovery,**19*(12), 839–859. 10.1038/s41573-020-0084-6 33077937 10.1038/s41573-020-0084-6 PMC7721651

[26] Rees, H. A., & Liu, D. R. (2018). Base editing: Precision chemistry on the genome and transcriptome of living cells. *Nature Reviews Genetics,**19*(12), 770–788. 10.1038/s41576-018-0059-1 30341440 10.1038/s41576-018-0059-1

[27] Chen, L., Zhu, B., Ru, G., Meng, H., Yan, Y., Hong, M., Zhang, D., Luan, C., Zhang, S., Wu, H., Gao, H., Bai, S., Li, C., Ding, R., Xue, N., Lei, Z., Chen, Y., Guan, Y., Siwko, S., … Li, D. (2023). Re-engineering the adenine deaminase TadA-8e for efficient and specific CRISPR-based cytosine base editing. *Nature Biotechnology,**41*(5), 663–672. 10.1038/s41587-022-01532-7 36357717 10.1038/s41587-022-01532-7

[28] Neugebauer, M. E., Hsu, A., Arbab, M., Krasnow, N. A., McElroy, A. N., Pandey, S., Doman, J. L., Huang, T. P., Raguram, A., Banskota, S., Newby, G. A., Tolar, J., Osborn, M. J., & Liu, D. R. (2023). Evolution of an adenine base editor into a small, efficient cytosine base editor with low off-target activity. *Nature Biotechnology,**41*(5), 673–685. 10.1038/s41587-022-01533-6 36357719 10.1038/s41587-022-01533-6 PMC10188366

[29] Huang, J., Lin, Q., Fei, H., He, Z., Xu, H., Li, Y., Qu, K., Han, P., Gao, Q., Li, B., Liu, G., Zhang, L., Hu, J., Zhang, R., Zuo, E., Luo, Y., Ran, Y., Qiu, J. L., Zhao, K. T., & Gao, C. (2023). Discovery of deaminase functions by structure-based protein clustering. *Cell,**186*(15), 3182-3195 e14. 10.1016/j.cell.2023.05.041 37379837 10.1016/j.cell.2023.05.041

[30] Ran, F. A., Cong, L., Yan, W. X., Scott, D. A., Gootenberg, J. S., Kriz, A. J., Zetsche, B., Shalem, O., Wu, X., Makarova, K. S., Koonin, E. V., Sharp, P. A., & Zhang, F. (2015). In vivo genome editing using Staphylococcus aureus Cas9. *Nature,**520*(7546), 186–191. 10.1038/nature14299 25830891 10.1038/nature14299 PMC4393360

[31] Edraki, A., Mir, A., Ibraheim, R., Gainetdinov, I., Yoon, Y., Song, C. Q., Cao, Y., Gallant, J., Xue, W., Rivera-Perez, J. A., & Sontheimer, E. J. (2019). A compact, high-accuracy Cas9 with a dinucleotide PAM for in vivo genome editing. *Molecular Cell,**73*(4), 714-726 e4. 10.1016/j.molcel.2018.12.003 30581144 10.1016/j.molcel.2018.12.003 PMC6386616

[32] Richter, M. F., Zhao, K. T., Eton, E., Lapinaite, A., Newby, G. A., Thuronyi, B. W., Wilson, C., Koblan, L. W., Zeng, J., Bauer, D. E., Doudna, J. A., & Liu, D. R. (2020). Phage-assisted evolution of an adenine base editor with improved Cas domain compatibility and activity. *Nature Biotechnology,**38*(7), 883–891. 10.1038/s41587-020-0453-z 32433547 10.1038/s41587-020-0453-z PMC7357821

[33] Kim, Y. B., Komor, A. C., Levy, J. M., Packer, M. S., Zhao, K. T., & Liu, D. R. (2017). Increasing the genome-targeting scope and precision of base editing with engineered Cas9-cytidine deaminase fusions. *Nature Biotechnology,**35*(4), 371–376. 10.1038/nbt.3803 28191901 10.1038/nbt.3803 PMC5388574

[34] Hu, J. H., Miller, S. M., Geurts, M. H., Tang, W., Chen, L., Sun, N., Zeina, C. M., Gao, X., Rees, H. A., Lin, Z., & Liu, D. R. (2018). Evolved Cas9 variants with broad PAM compatibility and high DNA specificity. *Nature,**556*(7699), 57–63. 10.1038/nature26155 29512652 10.1038/nature26155 PMC5951633

[35] Kleinstiver, B. P., Prew, M. S., Tsai, S. Q., Topkar, V. V., Nguyen, N. T., Zheng, Z., Gonzales, A. P., Li, Z., Peterson, R. T., Yeh, J. R., Aryee, M. J., & Joung, J. K. (2015). Engineered CRISPR-Cas9 nucleases with altered PAM specificities. *Nature,**523*(7561), 481–485. 10.1038/nature14592 26098369 10.1038/nature14592 PMC4540238

[36] Goldberg, G. W., Spencer, J. M., Giganti, D. O., Camellato, B. R., Agmon, N., Ichikawa, D. M., Boeke, J. D., & Noyes, M. B. (2021). Engineered dual selection for directed evolution of SpCas9 PAM specificity. *Nature Communications,**12*(1), 349. 10.1038/s41467-020-20650-x 33441553 10.1038/s41467-020-20650-x PMC7807044

[37] Landrum, M. J., Lee, J. M., Riley, G. R., Jang, W., Rubinstein, W. S., Church, D. M., & Maglott, D. R. (2014). ClinVar: Public archive of relationships among sequence variation and human phenotype. *Nucleic Acids Research,**42*(Database issue), D980–D985. 10.1093/nar/gkt1113 24234437 10.1093/nar/gkt1113 PMC3965032

[38] Koblan, L. W., Doman, J. L., Wilson, C., Levy, J. M., Tay, T., Newby, G. A., Maianti, J. P., Raguram, A., & Liu, D. R. (2018). Improving cytidine and adenine base editors by expression optimization and ancestral reconstruction. *Nature Biotechnology,**36*(9), 843–846. 10.1038/nbt.4172 29813047 10.1038/nbt.4172 PMC6126947

[39] Kurt, I. C., Zhou, R., Iyer, S., Garcia, S. P., Miller, B. R., Langner, L. M., Grunewald, J., & Joung, J. K. (2021). CRISPR C-to-G base editors for inducing targeted DNA transversions in human cells. *Nature Biotechnology,**39*(1), 41–46. 10.1038/s41587-020-0609-x 32690971 10.1038/s41587-020-0609-x PMC7854778

[40] Filippi, M. D., & Ghaffari, S. (2019). Mitochondria in the maintenance of hematopoietic stem cells: New perspectives and opportunities. *Blood,**133*(18), 1943–1952. 10.1182/blood-2018-10-808873 30808633 10.1182/blood-2018-10-808873 PMC6497515

[41] Ahlqvist, K. J., Leoncini, S., Pecorelli, A., Wortmann, S. B., Ahola, S., Forsstrom, S., Guerranti, R., De Felice, C., Smeitink, J., Ciccoli, L., Hamalainen, R. H., & Suomalainen, A. (2015). MtDNA mutagenesis impairs elimination of mitochondria during erythroid maturation leading to enhanced erythrocyte destruction. *Nature Communications,**6*, 6494. 10.1038/ncomms7494 25751021 10.1038/ncomms7494

[42] Gattermann, N., Retzlaff, S., Wang, Y. L., Hofhaus, G., Heinisch, J., Aul, C., & Schneider, W. (1997). Heteroplasmic point mutations of mitochondrial DNA affecting subunit I of cytochrome c oxidase in two patients with acquired idiopathic sideroblastic anemia. *Blood, 90*(12), 4961–4972. 10.1182/blood.V90.12.4961.4961_4961_4972 9389715

[43] Mok, B. Y., de Moraes, M. H., Zeng, J., Bosch, D. E., Kotrys, A. V., Raguram, A., Hsu, F., Radey, M. C., Peterson, S. B., Mootha, V. K., Mougous, J. D., & Liu, D. R. (2020). A bacterial cytidine deaminase toxin enables CRISPR-free mitochondrial base editing. *Nature,**583*(7817), 631–637. 10.1038/s41586-020-2477-4 32641830 10.1038/s41586-020-2477-4 PMC7381381

[44] Cho, S. I., Lee, S., Mok, Y. G., Lim, K., Lee, J., Lee, J. M., Chung, E., & Kim, J. S. (2022). Targeted A-to-G base editing in human mitochondrial DNA with programmable deaminases. *Cell,**185*(10), 1764-1776 e12. 10.1016/j.cell.2022.03.039 35472302 10.1016/j.cell.2022.03.039

[45] Eaves, C. J. (2015). Hematopoietic stem cells: Concepts, definitions, and the new reality. *Blood,**125*(17), 2605–2613. 10.1182/blood-2014-12-570200 25762175 10.1182/blood-2014-12-570200 PMC4440889

[46] Morgan, R. A., Gray, D., Lomova, A., & Kohn, D. B. (2017). Hematopoietic stem cell gene therapy: Progress and lessons learned. *Cell Stem Cell,**21*(5), 574–590. 10.1016/j.stem.2017.10.010 29100011 10.1016/j.stem.2017.10.010 PMC6039108

[47] Kato, G. J., Piel, F. B., Reid, C. D., Gaston, M. H., Ohene-Frempong, K., Krishnamurti, L., Smith, W. R., Panepinto, J. A., Weatherall, D. J., Costa, F. F., & Vichinsky, E. P. (2018). Sickle cell disease. *Nature Reviews Disease Primers,**4*, 18010. 10.1038/nrdp.2018.10 29542687 10.1038/nrdp.2018.10

[48] Piel, F. B., Steinberg, M. H., & Rees, D. C. (2017). Sickle cell disease. *The New England Journal of Medicine,**376*(16), 1561–1573. 10.1056/NEJMra1510865 28423290 10.1056/NEJMra1510865

[49] Frangoul, H., Altshuler, D., Cappellini, M. D., Chen, Y. S., Domm, J., Eustace, B. K., Foell, J., De La Fuente, J., Grupp, S., Handgretinger, R., Ho, T. W., Kattamis, A., Kernytsky, A., Lekstrom-Himes, J., Li, A. M., Locatelli, F., Mapara, M. Y., De Montalembert, M., Rondelli, D., … Corbacioglu, S. (2021). CRISPR-Cas9 gene editing for sickle cell disease and beta-thalassemia. *The New England Journal of Medicine,**384*(3), 252–260. 10.1056/NEJMoa2031054 33283989 10.1056/NEJMoa2031054

[50] Kosicki, M., Tomberg, K., & Bradley, A. (2018). Repair of double-strand breaks induced by CRISPR-Cas9 leads to large deletions and complex rearrangements. *Nature Biotechnology,**36*(8), 765–771. 10.1038/nbt.4192 30010673 10.1038/nbt.4192 PMC6390938

[51] Boutin, J., Rosier, J., Cappellen, D., Prat, F., Toutain, J., Pennamen, P., Bouron, J., Rooryck, C., Merlio, J. P., Lamrissi-Garcia, I., Cullot, G., Amintas, S., Guyonnet-Duperat, V., Ged, C., Blouin, J. M., Richard, E., Dabernat, S., Moreau-Gaudry, F., & Bedel, A. (2021). CRISPR-Cas9 globin editing can induce megabase-scale copy-neutral losses of heterozygosity in hematopoietic cells. *Nature Communications, 12*(1). 10.1038/s41467-021-25190-6 10.1038/s41467-021-25190-6 PMC8363739 34389729

[52] Newby, G. A., Yen, J. S., Woodard, K. J., Mayuranathan, T., Lazzarotto, C. R., Li, Y., Sheppard-Tillman, H., Porter, S. N., Yao, Y., Mayberry, K., Everette, K. A., Jang, Y., Podracky, C. J., Thaman, E., Lechauve, C., Sharma, A., Henderson, J. M., Richter, M. F., Zhao, K. T., … Liu, D. R. (2021). Base editing of haematopoietic stem cells rescues sickle cell disease in mice. *Nature,**595*(7866), 295–302. 10.1038/s41586-021-03609-w 34079130 10.1038/s41586-021-03609-w PMC8266759

[53] Everette, K. A., Newby, G. A., Levine, R. M., Mayberry, K., Jang, Y., Mayuranathan, T., Nimmagadda, N., Dempsey, E., Li, Y., Bhoopalan, S. V., Liu, X., Davis, J. R., Nelson, A. T., Chen, P. J., Sousa, A. A., Cheng, Y., Tisdale, J. F., Weiss, M. J., Yen, J. S., & Liu, D. R. (2023). Ex vivo prime editing of patient haematopoietic stem cells rescues sickle-cell disease phenotypes after engraftment in mice. *Nature Biomedical Engineering,**7*(5), 616–628. 10.1038/s41551-023-01026-0 37069266 10.1038/s41551-023-01026-0 PMC10195679

[54] Cao, A., & Galanello, R. (2010). Beta-thalassemia. *Genetics in Medicine,**12*(2), 61–76. 10.1097/GIM.0b013e3181cd68ed 20098328 10.1097/GIM.0b013e3181cd68ed

[55] Taher, A. T., Weatherall, D. J., & Cappellini, M. D. (2018). Thalassaemia. *The Lancet,**391*(10116), 155–167. 10.1016/s0140-6736(17)31822-6 10.1016/s0140-6736(17)31822-6 28774421

[56] Hardouin, G., Antoniou, P., Martinucci, P., Felix, T., Manceau, S., Joseph, L., Masson, C., Scaramuzza, S., Ferrari, G., Cavazzana, M., & Miccio, A. (2023). Adenine base editor-mediated correction of the common and severe IVS1-110 (G>A) beta-thalassemia mutation. *Blood,**141*(10), 1169–1179. 10.1182/blood.2022016629 36508706 10.1182/blood.2022016629 PMC10651780

[57] Sebastiani, P., & Steinberg, M. H. (2022). Fetal hemoglobin per erythrocyte (HbF/F-cell) after gene therapy for sickle cell anemia. *American Journal of Hematology,**98*(2), E32–E34. 10.1002/ajh.26791 36420999 10.1002/ajh.26791

[58] Canver, M. C., Smith, E. C., Sher, F., Pinello, L., Sanjana, N. E., Shalem, O., Chen, D. D., Schupp, P. G., Vinjamur, D. S., Garcia, S. P., Luc, S., Kurita, R., Nakamura, Y., Fujiwara, Y., Maeda, T., Yuan, G. C., Zhang, F., Orkin, S. H., & Bauer, D. E. (2015). BCL11A enhancer dissection by Cas9-mediated in situ saturating mutagenesis. *Nature,**527*(7577), 192–197. 10.1038/nature15521 26375006 10.1038/nature15521 PMC4644101

[59] Wu, Y., Zeng, J., Roscoe, B. P., Liu, P., Yao, Q., Lazzarotto, C. R., Clement, K., Cole, M. A., Luk, K., Baricordi, C., Shen, A. H., Ren, C., Esrick, E. B., Manis, J. P., Dorfman, D. M., Williams, D. A., Biffi, A., Brugnara, C., Biasco, L., … Bauer, D. E. (2019). Highly efficient therapeutic gene editing of human hematopoietic stem cells. *Nature Medicine,**25*(5), 776–783. 10.1038/s41591-019-0401-y 30911135 10.1038/s41591-019-0401-y PMC6512986

[60] Zeng, J., Wu, Y., Ren, C., Bonanno, J., Shen, A. H., Shea, D., Gehrke, J. M., Clement, K., Luk, K., Yao, Q., Kim, R., Wolfe, S. A., Manis, J. P., Pinello, L., Joung, J. K., & Bauer, D. E. (2020). Therapeutic base editing of human hematopoietic stem cells. *Nature Medicine,**26*(4), 535–541. 10.1038/s41591-020-0790-y 32284612 10.1038/s41591-020-0790-y PMC7869435

[61] Mayuranathan, T., Newby, G. A., Feng, R., Yao, Y., Mayberry, K. D., Lazzarotto, C. R., Li, Y., Levine, R. M., Nimmagadda, N., Dempsey, E., Kang, G., Porter, S. N., Doerfler, P. A., Zhang, J., Jang, Y., Chen, J., Bell, H. W., Crossley, M., Bhoopalan, S. V., … Yen, J. S. (2023). Potent and uniform fetal hemoglobin induction via base editing. *Nature Genetics,**55*(7), 1210–1220. 10.1038/s41588-023-01434-7 37400614 10.1038/s41588-023-01434-7 PMC10722557

[62] Taniguchi, T., & D’Andrea, A. D. (2006). Molecular pathogenesis of Fanconi anemia: Recent progress. *Blood,**107*(11), 4223–4233. 10.1182/blood-2005-10-4240 16493006 10.1182/blood-2005-10-4240

[63] Siegner, S. M., Ugalde, L., Clemens, A., Garcia-Garcia, L., Bueren, J. A., Rio, P., Karasu, M. E., & Corn, J. E. (2022). Adenine base editing efficiently restores the function of Fanconi anemia hematopoietic stem and progenitor cells. *Nature Communications,**13*(1), 6900. 10.1038/s41467-022-34479-z 36371486 10.1038/s41467-022-34479-z PMC9653444

[64] Dadi, H. K., Simon, A. J., & Roifman, C. M. (2003). Effect of CD3delta deficiency on maturation of alpha/beta and gamma/delta T-cell lineages in severe combined immunodeficiency. *The New England Journal of Medicine,**349*(19), 1821–1828. 10.1056/NEJMoa031178 14602880 10.1056/NEJMoa031178

[65] McAuley, G. E., Yiu, G., Chang, P. C., Newby, G. A., Campo-Fernandez, B., Fitz-Gibbon, S. T., Wu, X., Kang, S. L., Garibay, A., Butler, J., Christian, V., Wong, R. L., Everette, K. A., Azzun, A., Gelfer, H., Seet, C. S., Narendran, A., Murguia-Favela, L., Romero, Z., … Kohn, D. B. (2023). Human T cell generation is restored in CD3delta severe combined immunodeficiency through adenine base editing. *Cell,**186*(7), 1398-1416.e23. 10.1016/j.cell.2023.02.027 36944331 10.1016/j.cell.2023.02.027 PMC10876291

[66] Raguram, A., Banskota, S., & Liu, D. R. (2022). Therapeutic in vivo delivery of gene editing agents. *Cell,**185*(15), 2806–2827. 10.1016/j.cell.2022.03.045 35798006 10.1016/j.cell.2022.03.045 PMC9454337

[67] Lee, C. S., Bishop, E. S., Zhang, R., Yu, X., Farina, E. M., Yan, S., Zhao, C., Zheng, Z., Shu, Y., Wu, X., Lei, J., Li, Y., Zhang, W., Yang, C., Wu, K., Wu, Y., Ho, S., Athiviraham, A., Lee, M. J., … He, T. C. (2017). Adenovirus-mediated gene delivery: Potential applications for gene and cell-based therapies in the new era of personalized medicine. *Genes & Diseases,**4*(2), 43–63. 10.1016/j.gendis.2017.04.001 28944281 10.1016/j.gendis.2017.04.001 PMC5609467

[68] Cots, D., Bosch, A., & Chillon, M. (2013). Helper dependent adenovirus vectors: Progress and future prospects. *Current Gene Therapy,**13*(5), 370–381. 10.2174/156652321305131212125338 24369061 10.2174/156652321305131212125338

[69] Richter, M., Saydaminova, K., Yumul, R., Krishnan, R., Liu, J., Nagy, E. E., Singh, M., Izsvak, Z., Cattaneo, R., Uckert, W., Palmer, D., Ng, P., Haworth, K. G., Kiem, H. P., Ehrhardt, A., Papayannopoulou, T., & Lieber, A. (2016). In vivo transduction of primitive mobilized hematopoietic stem cells after intravenous injection of integrating adenovirus vectors. *Blood,**128*(18), 2206–2217. 10.1182/blood-2016-04-711580 27554082 10.1182/blood-2016-04-711580 PMC5095755

[70] Breda, L., Papp, T. E., Triebwasser, M. P., Yadegari, A., Fedorky, M. T., Tanaka, N., Abdulmalik, O., Pavani, G., Wang, Y., Grupp, S. A., Chou, S. T., Ni, H., Mui, B. L., Tam, Y. K., Weissman, D., Rivella, S., & Parhiz, H. (2023). In vivo hematopoietic stem cell modification by mRNA delivery. *Science,**381*(6656), 436–443. 10.1126/science.ade6967 37499029 10.1126/science.ade6967 PMC10567133

[71] Li, C., Georgakopoulou, A., Mishra, A., Gil, S., Hawkins, R. D., Yannaki, E., & Lieber, A. (2021). In vivo HSPC gene therapy with base editors allows for efficient reactivation of fetal gamma-globin in beta-YAC mice. *Blood Advances,**5*(4), 1122–1135. 10.1182/bloodadvances.2020003702 33620431 10.1182/bloodadvances.2020003702 PMC7903237

[72] Li, C., Georgakopoulou, A., Newby, G. A., Everette, K. A., Nizamis, E., Paschoudi, K., Vlachaki, E., Gil, S., Anderson, A. K., Koob, T., Huang, L., Wang, H., Kiem, H. P., Liu, D. R., Yannaki, E., & Lieber, A. (2022). In vivo base editing by a single i.v. vector injection for treatment of hemoglobinopathies. *JCI Insight,**7*(19), e162939. 10.1172/jci.insight.162939 36006707 10.1172/jci.insight.162939 PMC9675455

[73] Li, C., Georgakopoulou, A., Newby, G. A., Chen, P. J., Everette, K. A., Paschoudi, K., Vlachaki, E., Gil, S., Anderson, A. K., Koob, T., Huang, L., Wang, H., Kiem, H. P., Liu, D. R., Yannaki, E., & Lieber, A. (2023). In vivo HSC prime editing rescues sickle cell disease in a mouse model. *Blood,**141*(17), 2085–2099. 10.1182/blood.2022018252 36800642 10.1182/blood.2022018252 PMC10163316

[74] Charlesworth, C. T., Hsu, I., Wilkinson, A. C., & Nakauchi, H. (2022). Immunological barriers to haematopoietic stem cell gene therapy. *Nature Reviews Immunology,**22*(12), 719–733. 10.1038/s41577-022-00698-0 35301483 10.1038/s41577-022-00698-0 PMC8929255

[75] Harteveld, C. L., & Higgs, D. R. (2010). Alpha-thalassaemia. *Orphanet Journal of Rare Diseases,**5*, 13. 10.1186/1750-1172-5-13 20507641 10.1186/1750-1172-5-13 PMC2887799

[76] King, A. J., Songdej, D., Downes, D. J., Beagrie, R. A., Liu, S., Buckley, M., Hua, P., Suciu, M. C., Oudelaar, A. M., Hanssen, L. L. P., Jeziorska, D., Roberts, N., Carpenter, S. J., Francis, H., Telenius, J., Olijnik, A. A., Sharpe, J. A., Sloane-Stanley, J., Eglinton, J., … Babbs, C. (2021). Reactivation of a developmentally silenced embryonic globin gene. *Nature Communications,**12*(1), 4439. 10.1038/s41467-021-24402-3 34290235 10.1038/s41467-021-24402-3 PMC8295333

[77] Li, L., Yi, H., Liu, Z., Long, P., Pan, T., Huang, Y., Li, Y., Li, Q., & Ma, Y. (2022). Genetic correction of concurrent alpha- and beta-thalassemia patient-derived pluripotent stem cells by the CRISPR-Cas9 technology. *Stem Cell Research & Therapy,**13*(1), 102. 10.1186/s13287-022-02768-5 35255977 10.1186/s13287-022-02768-5 PMC8900422

[78] Narla, A., & Ebert, B. L. (2010). Ribosomopathies: Human disorders of ribosome dysfunction. *Blood,**115*(16), 3196–3205. 10.1182/blood-2009-10-178129 20194897 10.1182/blood-2009-10-178129 PMC2858486

[79] O’Donohue, M. F., Da Costa, L., Lezzerini, M., Unal, S., Joret, C., Bartels, M., Brilstra, E., Scheijde-Vermeulen, M., Wacheul, L., De Keersmaecker, K., Vereecke, S., Labarque, V., Saby, M., Lefevre, S. D., Platon, J., Montel-Lehry, N., Laugero, N., Lacazette, E., van Gassen, K., … MacInnes, A. W. (2022). HEATR3 variants impair nuclear import of uL18 (RPL5) and drive Diamond-Blackfan anemia. *Blood,**139*(21), 3111–3126. 10.1182/blood.2021011846 35213692 10.1182/blood.2021011846 PMC9136880

[80] Vlachos, A., Federman, N., Reyes-Haley, C., Abramson, J., & Lipton, J. M. (2001). Hematopoietic stem cell transplantation for Diamond Blackfan anemia: A report from the Diamond Blackfan anemia registry. *Bone Marrow Transplantation,**27*(4), 381–386. 10.1038/sj.bmt.1702784 11313667 10.1038/sj.bmt.1702784

[81] Naseem, A., Steinberg, Z., & Cavazza, A. (2022). Genome editing for primary immunodeficiencies: A therapeutic perspective on Wiskott-Aldrich syndrome. *Frontiers in Immunology,**13*, 966084. 10.3389/fimmu.2022.966084 36059471 10.3389/fimmu.2022.966084 PMC9433875

[82] Jin, Y., Mazza, C., Christie, J. R., Giliani, S., Fiorini, M., Mella, P., Gandellini, F., Stewart, D. M., Zhu, Q., Nelson, D. L., Notarangelo, L. D., & Ochs, H. D. (2004). Mutations of the Wiskott-Aldrich syndrome protein (WASP): Hotspots, effect on transcription, and translation and phenotype/genotype correlation. *Blood,**104*(13), 4010–4019. 10.1182/blood-2003-05-1592 15284122 10.1182/blood-2003-05-1592

[83] Rai, R., Romito, M., Rivers, E., Turchiano, G., Blattner, G., Vetharoy, W., Ladon, D., Andrieux, G., Zhang, F., Zinicola, M., Leon-Rico, D., Santilli, G., Thrasher, A. J., & Cavazza, A. (2020). Targeted gene correction of human hematopoietic stem cells for the treatment of Wiskott - Aldrich syndrome. *Nature Communications,**11*(1), 4034. 10.1038/s41467-020-17626-2 32788576 10.1038/s41467-020-17626-2 PMC7423939

[84] Fischer, A., Le Deist, F., Hacein-Bey-Abina, S., Andre-Schmutz, I., Basile Gde, S., De Villartay, J. P., & Cavazzana-Calvo, M. (2005). Severe combined immunodeficiency. A model disease for molecular immunology and therapy. *Immunological Reviews,**203*, 98–109. 10.1111/j.0105-2896.2005.00223.x 15661024 10.1111/j.0105-2896.2005.00223.x

[85] Puck, J. M., Pepper, A. E., Henthorn, P. S., Candotti, F., Isakov, J., Whitwam, T., Conley, M. E., Fischer, R. E., Rosenblatt, H. M., Small, T. N., & Buckley, R. H. (1997). Mutation analysis of IL2RG in human X-linked severe combined immunodeficiency. *Blood,**89*, 1968–1977. 10.1182/blood.V89.6.1968 9058718 10.1182/blood.V89.6.1968

[86] Byambaa, S., Uosaki, H., Ohmori, T., Hara, H., Endo, H., Nureki, O., & Hanazono, Y. (2021). Non-viral ex vivo genome-editing in mouse bona fide hematopoietic stem cells with CRISPR/Cas9. *Molecular Therapy Methods & Clinical Development,**20*, 451–462. 10.1016/j.omtm.2021.01.001 33614821 10.1016/j.omtm.2021.01.001 PMC7873578

[87] Moreau, T., Calmels, B., Barlogis, V., Michel, G., Tonnelle, C., & Chabannon, C. (2007). Potential application of gene therapy to X-linked agammaglobulinemia. *Current Gene Therapy,**7*(4), 284–294. 10.2174/156652307781369128 17969561 10.2174/156652307781369128

[88] Wang, Y., Kanegane, H., Wang, X., Han, X., Zhang, Q., Zhao, S., Yu, Y., Wang, J., & Miyawaki, T. (2009). Mutation of the BTK gene and clinical feature of X-linked agammaglobulinemia in mainland China. *Journal of Clinical Immunology,**29*(3), 352–356. 10.1007/s10875-008-9262-8 19039656 10.1007/s10875-008-9262-8

[89] Stirnemann, J., Belmatoug, N., Camou, F., Serratrice, C., Froissart, R., Caillaud, C., Levade, T., Astudillo, L., Serratrice, J., Brassier, A., Rose, C., De Villemeur, T. B., & Berger, M. G. (2017). A review of Gaucher disease pathophysiology, clinical presentation and treatments. *International Journal of Molecular Sciences,**18*(2), 441. 10.3390/ijms18020441 28218669 10.3390/ijms18020441 PMC5343975

[90] Scharenberg, S. G., Poletto, E., Lucot, K. L., Colella, P., Sheikali, A., Montine, T. J., Porteus, M. H., & Gomez-Ospina, N. (2020). Engineering monocyte/macrophage-specific glucocerebrosidase expression in human hematopoietic stem cells using genome editing. *Nature Communications,**11*(1), 3327. 10.1038/s41467-020-17148-x 32620863 10.1038/s41467-020-17148-x PMC7335164

[91] Horowitz, M., & Zimran, A. (1994). Mutations causing Gaucher disease. *Human Mutation,**3*(1), 1–11. 10.1002/humu.1380030102 8118460 10.1002/humu.1380030102

[92] Rao, X., Zhao, H., Shao, C., & Yi, C. (2023). Characterizing off-target effects of genome editors. *Current Opinion in Biomedical Engineering,**28*, 100480. 10.1016/j.cobme.2023.100480 10.1016/j.cobme.2023.100480

[93] Park, S., & Beal, P. A. (2019). Off-target editing by CRISPR-guided DNA base editors. *Biochemistry,**58*(36), 3727–3734. 10.1021/acs.biochem.9b00573 31433621 10.1021/acs.biochem.9b00573 PMC7309633

[94] Pacesa, M., Lin, C. H., Clery, A., Saha, A., Arantes, P. R., Bargsten, K., Irby, M. J., Allain, F. H., Palermo, G., Cameron, P., Donohoue, P. D., & Jinek, M. (2022). Structural basis for Cas9 off-target activity. *Cell,**185*(22), 4067-4081.e21. 10.1016/j.cell.2022.09.026 36306733 10.1016/j.cell.2022.09.026 PMC10103147

[95] Rees, H. A., Komor, A. C., Yeh, W. H., Caetano-Lopes, J., Warman, M., Edge, A. S. B., & Liu, D. R. (2017). Improving the DNA specificity and applicability of base editing through protein engineering and protein delivery. *Nature Communications,**8*, 15790. 10.1038/ncomms15790 28585549 10.1038/ncomms15790 PMC5467206

[96] Lee, J. K., Jeong, E., Lee, J., Jung, M., Shin, E., Kim, Y. H., Lee, K., Jung, I., Kim, D., Kim, S., & Kim, J. S. (2018). Directed evolution of CRISPR-Cas9 to increase its specificity. *Nature Communications,**9*(1), 3048. 10.1038/s41467-018-05477-x 30082838 10.1038/s41467-018-05477-x PMC6078992

[97] Zuo, E., Sun, Y., Wei, W., Yuan, T., Ying, W., Sun, H., Yuan, L., Steinmetz, L. M., Li, Y., & Yang, H. (2019). Cytosine base editor generates substantial off-target single-nucleotide variants in mouse embryos. *Science,**364*(6437), 289–292. 10.1126/science.aav9973 30819928 10.1126/science.aav9973 PMC7301308

[98] Jin, S., Zong, Y., Gao, Q., Zhu, Z. X., Wang, Y. P., Qin, P., Liang, C. Z., Wang, D. W., Qiu, J. L., Zhang, F., & Gao, C. X. (2019). Cytosine, but not adenine, base editors induce genome-wide off-target mutations in rice. *Science,**364*(6437), 292–295. 10.1126/science.aaw7166 30819931 10.1126/science.aaw7166

[99] Doman, J. L., Raguram, A., Newby, G. A., & Liu, D. R. (2020). Evaluation and minimization of Cas9-independent off-target DNA editing by cytosine base editors. *Nature Biotechnology,**38*(5), 620–628. 10.1038/s41587-020-0414-6 32042165 10.1038/s41587-020-0414-6 PMC7335424

[100] Yu, Y., Leete, T. C., Born, D. A., Young, L., Barrera, L. A., Lee, S. J., Rees, H. A., Ciaramella, G., & Gaudelli, N. M. (2020). Cytosine base editors with minimized unguided DNA and RNA off-target events and high on-target activity. *Nature Communications,**11*(1), 2052. 10.1038/s41467-020-15887-5 32345976 10.1038/s41467-020-15887-5 PMC7189382

[101] Yuan, B., Zhang, S., Song, L., Chen, J., Cao, J., Qiu, J., Qiu, Z., Chen, J., Zhao, X. M., & Cheng, T. L. (2023). Engineering of cytosine base editors with DNA damage minimization and editing scope diversification. *Nucleic Acids Research,**51*(20), e105. 10.1093/nar/gkad855 37843111 10.1093/nar/gkad855 PMC10639057

[102] Zhang, S., Song, L., Yuan, B., Zhang, C., Cao, J., Chen, J., Qiu, J., Tai, Y., Chen, J., Qiu, Z., Zhao, X. M., & Cheng, T. L. (2023). TadA reprogramming to generate potent miniature base editors with high precision. *Nature Communications,**14*(1), 413. 10.1038/s41467-023-36004-2 36702845 10.1038/s41467-023-36004-2 PMC9879996

[103] Grünewald, J., Zhou, R., Garcia, S. P., Iyer, S., Lareau, C. A., Aryee, M. J., & Joung, J. K. (2019). Transcriptome-wide off-target RNA editing induced by CRISPR-guided DNA base editors. *Nature,**569*(7756), 433–437. 10.1038/s41586-019-1161-z 30995674 10.1038/s41586-019-1161-z PMC6657343

[104] Grunewald, J., Zhou, R., Iyer, S., Lareau, C. A., Garcia, S. P., Aryee, M. J., & Joung, J. K. (2019). CRISPR DNA base editors with reduced RNA off-target and self-editing activities. *Nature Biotechnology,**37*(9), 1041–1048. 10.1038/s41587-019-0236-6 31477922 10.1038/s41587-019-0236-6 PMC6730565

[105] Fiumara, M., Ferrari, S., Omer-Javed, A., Beretta, S., Albano, L., Canarutto, D., Varesi, A., Gaddoni, C., Brombin, C., Cugnata, F., Zonari, E., Naldini, M. M., Barcella, M., Gentner, B., Merelli, I., & Naldini, L. (2023). Genotoxic effects of base and prime editing in human hematopoietic stem cells. *Nature Biotechnology*. 10.1038/s41587-023-01915-4 37679541 10.1038/s41587-023-01915-4 PMC11180610

[106] Yan, N., Feng, H., Sun, Y., Xin, Y., Zhang, H., Lu, H., Zheng, J., He, C., Zuo, Z., Yuan, T., Li, N., Xie, L., Wei, W., Sun, Y., & Zuo, E. (2023). Cytosine base editors induce off-target mutations and adverse phenotypic effects in transgenic mice. *Nature Communications,**14*(1), 1784. 10.1038/s41467-023-37508-7 36997536 10.1038/s41467-023-37508-7 PMC10063651

[107] Han, W., Qiu, H. Y., Sun, S., Fu, Z. C., Wang, G. Q., Qian, X., Wang, L., Zhai, X., Wei, J., Wang, Y., Guo, Y. L., Cao, G. H., Ji, R. J., Zhang, Y. Z., Ma, H., Wang, H., Zhao, M., Wu, J., Bi, L., … Zhang, Y. (2023). Base editing of the HBG promoter induces potent fetal hemoglobin expression with no detectable off-target mutations in human HSCs. *Cell Stem Cell,**30*(12), 1624-1639 e8. 10.1016/j.stem.2023.10.007 37989316 10.1016/j.stem.2023.10.007

[108] Walton, R. T., Christie, K. A., Whittaker, M. N., & Kleinstiver, B. P. (2020). Unconstrained genome targeting with near-PAMless engineered CRISPR-Cas9 variants. *Science,**368*(6488), 290–296. 10.1126/science.aba8853 32217751 10.1126/science.aba8853 PMC7297043

[109] Gehrke, J. M., Cervantes, O., Clement, M. K., Wu, Y., Zeng, J., Bauer, D. E., Pinello, L., & Joung, J. K. (2018). An APOBEC3A-Cas9 base editor with minimized bystander and off-target activities. *Nature Biotechnology,**36*(10), 977–982. 10.1038/nbt.4199 30059493 10.1038/nbt.4199 PMC6181770

[110] Becker, H. J., Ishida, R., Wilkinson, A. C., Kimura, T., Lee, M. S. J., Coban, C., Ota, Y., Tanaka, Y., Roskamp, M., Sano, T., Tojo, A., Kent, D. G., & Yamazaki, S. (2023). Controlling genetic heterogeneity in gene-edited hematopoietic stem cells by single-cell expansion. *Cell Stem Cell,**30*(7), 987-1000 e8. 10.1016/j.stem.2023.06.002 37385251 10.1016/j.stem.2023.06.002 PMC10338855

[111] Chen, L., Hong, M., Luan, C., Gao, H., Ru, G., Guo, X., Zhang, D., Zhang, S., Li, C., Wu, J., Randolph, P. B., Sousa, A. A., Qu, C., Zhu, Y., Guan, Y., Wang, L., Liu, M., Feng, B., Song, G., … Li, D. (2023). Adenine transversion editors enable precise, efficient A*T-to-C*G base editing in mammalian cells and embryos. *Nature Biotechnology*. 10.1038/s41587-023-01821-9 37735269 10.1038/s41587-023-01821-9

[112] Yuan, T., Yan, N., Fei, T., Zheng, J., Meng, J., Li, N., Liu, J., Zhang, H., Xie, L., Ying, W., Li, D., Shi, L., Sun, Y., Li, Y., Li, Y., Sun, Y., & Zuo, E. (2021). Optimization of C-to-G base editors with sequence context preference predictable by machine learning methods. *Nature Communications,**12*(1), 4902. 10.1038/s41467-021-25217-y 34385461 10.1038/s41467-021-25217-y PMC8361092

[113] Marquart, K. F., Allam, A., Janjuha, S., Sintsova, A., Villiger, L., Frey, N., Krauthammer, M., & Schwank, G. (2021). Predicting base editing outcomes with an attention-based deep learning algorithm trained on high-throughput target library screens. *Nature Communications,**12*(1), 5114. 10.1038/s41467-021-25375-z 34433819 10.1038/s41467-021-25375-z PMC8387386

[114] Kim, N., Choi, S., Kim, S., Song, M., Seo, J. H., Min, S., Park, J., Cho, S. R., & Kim, H. H. (2023). Deep learning models to predict the editing efficiencies and outcomes of diverse base editors. *Nature Biotechnology*. 10.1038/s41587-023-01792-x 37188916 10.1038/s41587-023-01792-x

[115] Zhang, C., Yang, Y., Qi, T., Zhang, Y., Hou, L., Wei, J., Yang, J., Shi, L., Ong, S. G., Wang, H., Wang, H., Yu, B., & Wang, Y. (2023). Prediction of base editor off-targets by deep learning. *Nature Communications,**14*(1), 5358. 10.1038/s41467-023-41004-3 37660097 10.1038/s41467-023-41004-3 PMC10475126

